# Supplementary material for: Femtosecond Visible Transient Absorption Spectroscopy of Chlorophyll f-Containing Photosystem I
Source: Biophys J. 2017 Jan 24;112(2):234–49. doi: 10.1016/j.bpj.2016.12.022 (PMC5266252; doi:10.1016/j.bpj.2016.12.022)
Supplement: Document S1. Supporting Materials and Methods, Supporting Results, and Figs. S1–S6 [file mmc1.pdf]

Biophysical Journal, Volume *112*

Supplemental Information

Femtosecond Visible Transient Absorption Spectroscopy of Chlorophyll *f*-Containing Photosystem I

Marius Kaucikas, Dennis Nürnberg, Gabriel Dorlhiac, A. William Rutherford, and Jasper J. van Thor

Femtosecond visible transient absorption spectroscopy of Chlorophyll f-containing Photosystem I

M. Kaucikas, D. Nürnberg , G. Dorlhiac , A. W. Rutherford , J. J. van Thor

Supplementary information

Contents

Different Models for WL PSI with 670 nm excitation 2
Model 1: Sequential model 2
Model 2: Sequential model with reverse reactions 2
Model 3 2
Model 4 3
Model 5 3
Model 6 4
Model 7 4
The Effect of the initial excitation distribution between the compartments 4
Model 1: Sequential model 4
Model 3 5
Model 5 5
Fits for the WL PSI 700nm and FRL data 6
WL PSI 700 nm sequential 6
FRL PSI 670 nm excitation 6
Model FRL-1 6
Model FRL-2 7
Pump energy and sample translation effects 8
Theoretical calculations 8
Experimental tests 8
Estimation of Charge Separation Efficiency 13
References 14

Below we present several models that were applied to the PSI TA data. Model scheme, concentration profiles and SADS are shown for each model. The numbers in the model schemes indicates the rate in (ps^{-1}). If not indicated otherwise, the model assumes that initial excitation is compartment A.

Different Models for WL PSI with 670 nm excitation

Model 1: Sequential model

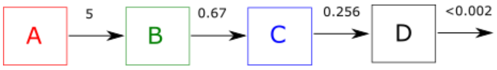

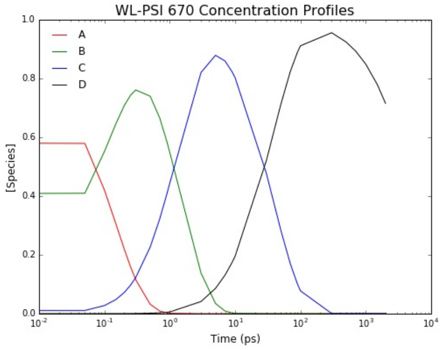

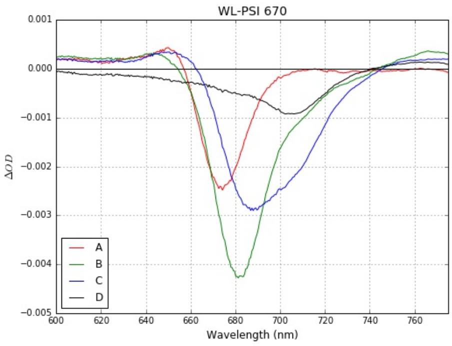

Model 2: Sequential model with reverse reactions

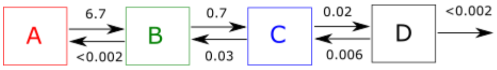

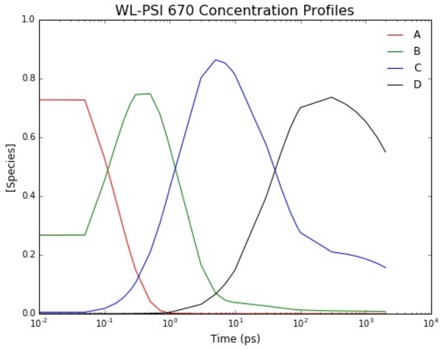

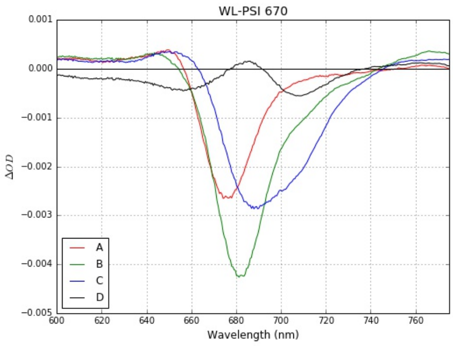

Model 3

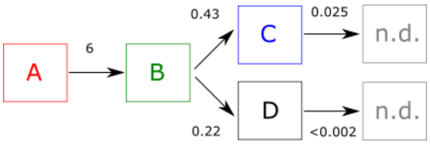

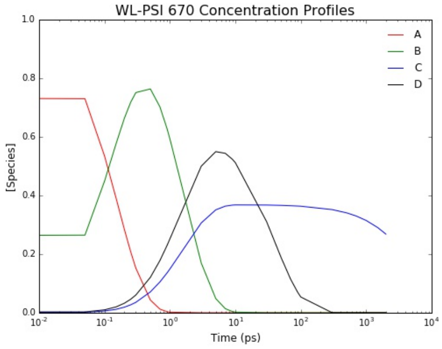

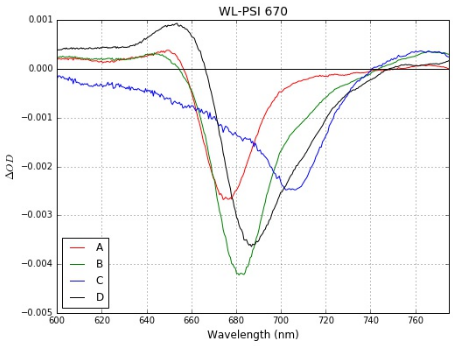

Model 4

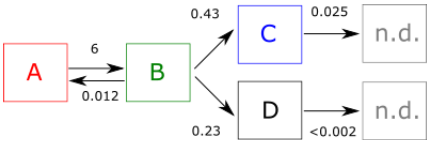

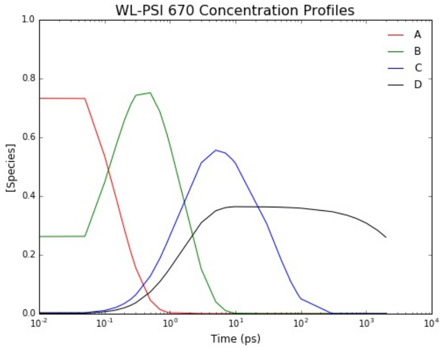

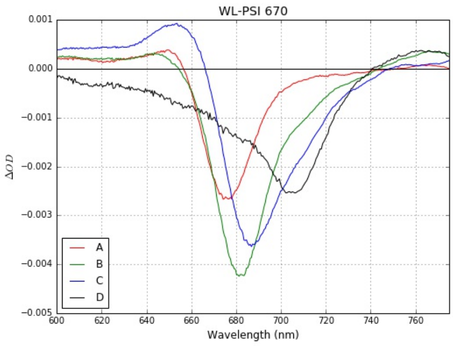

Model 5

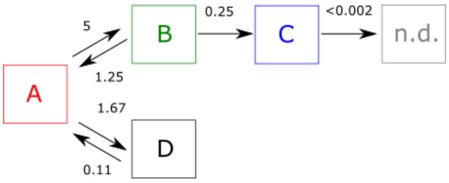

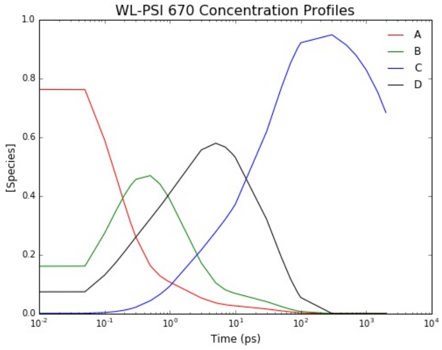

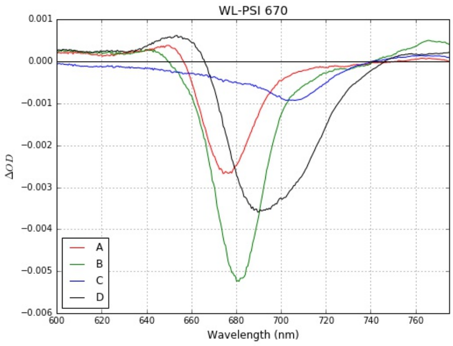

Model 6

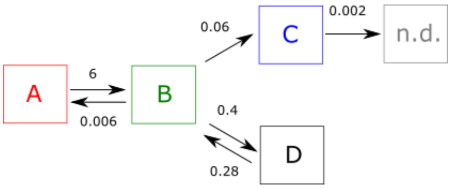

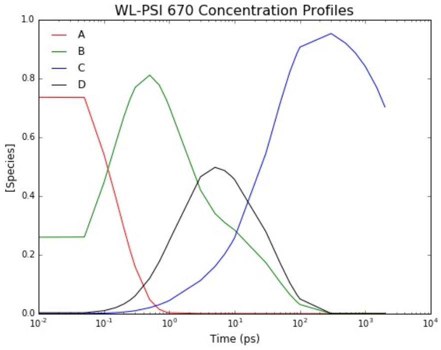

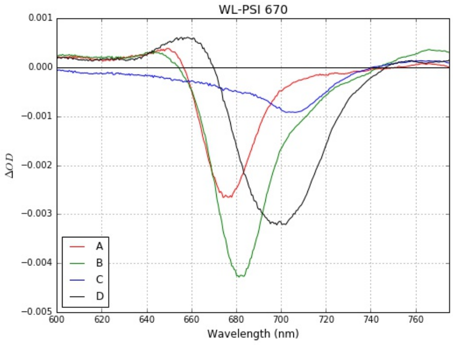

Model 7

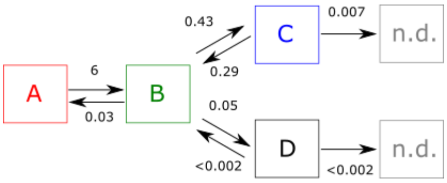

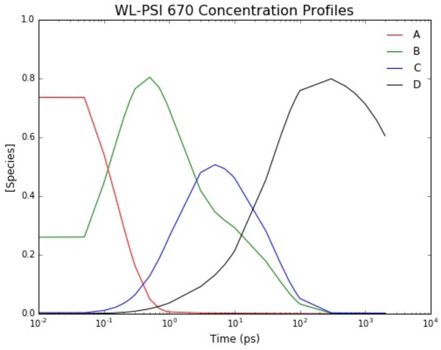

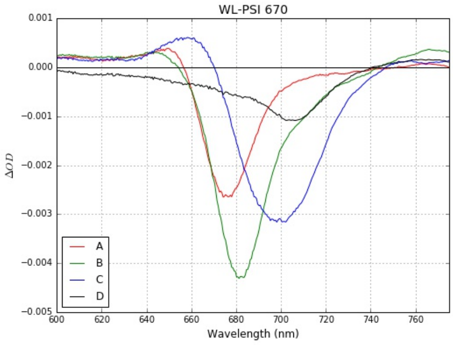

The Effect of the initial excitation distribution between the compartments

Model 1: Sequential model

Initial distribution: 80% A, 20% B

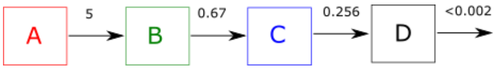

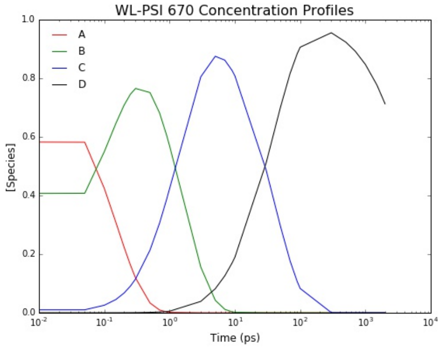

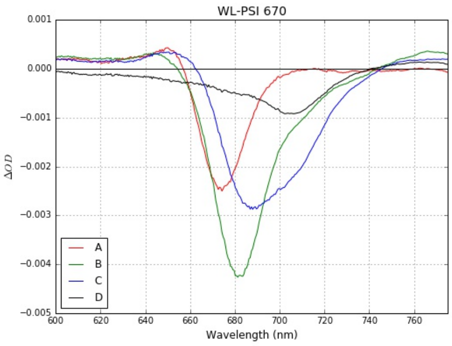

Model 3

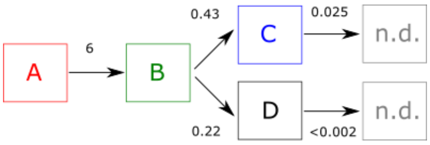

Initial distribution: 80% A, 20% B

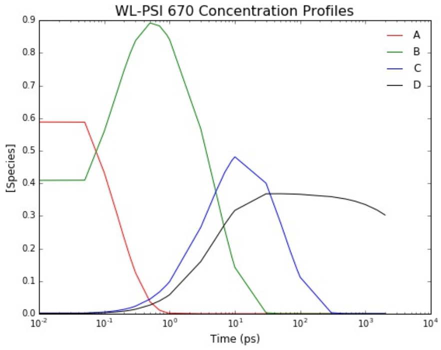

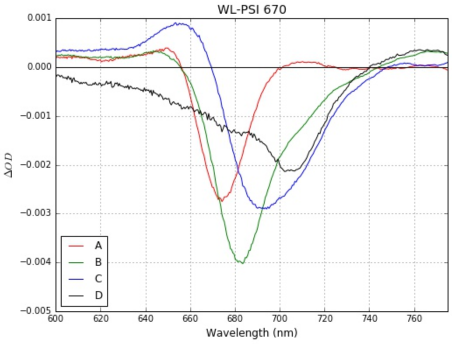

Model 5

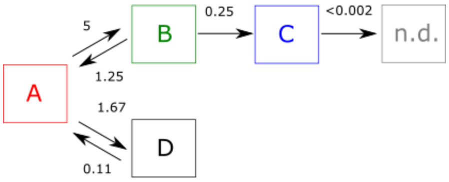

Initial distribution: 80% A, 20% B

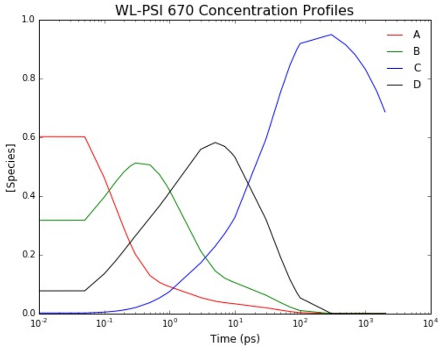

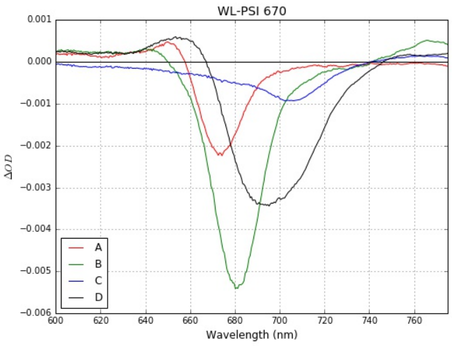

Initial distribution: 45% A, 25% B, 25% D

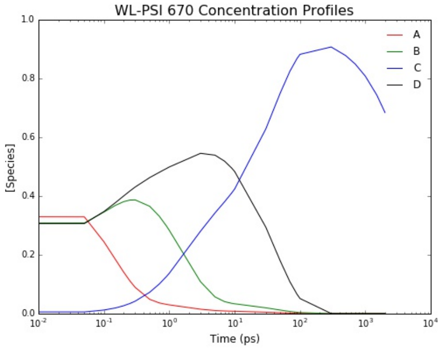

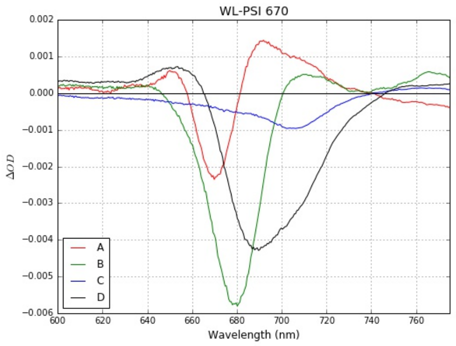

Fits for the WL PSI 700nm and FRL data

WL PSI 700 nm sequential

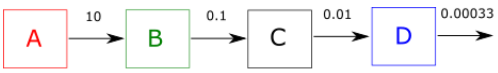

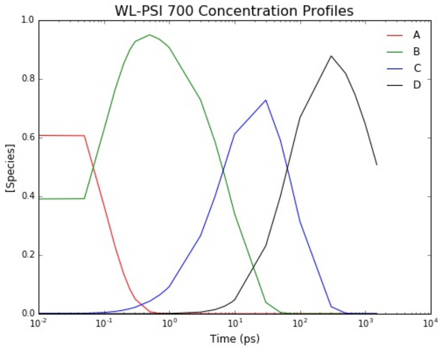

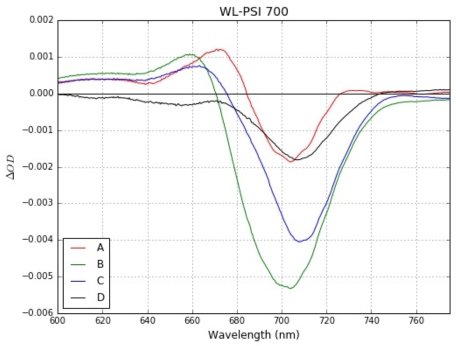

FRL PSI 670 nm excitation

Model FRL-1

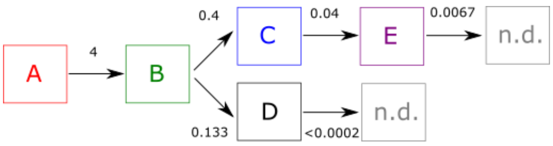

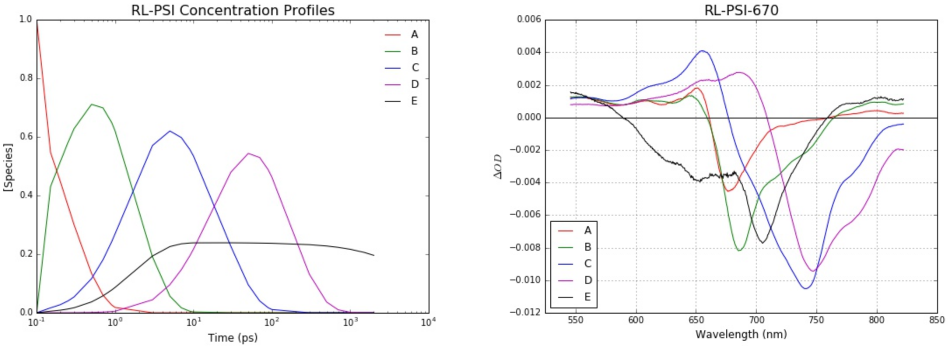
RL-PSI Concentration Profiles
[Species]
Time (ps)
A
B
C
D
E
RL-PSI-670
ΔOD
Wavelength (nm)

Model FRL-2

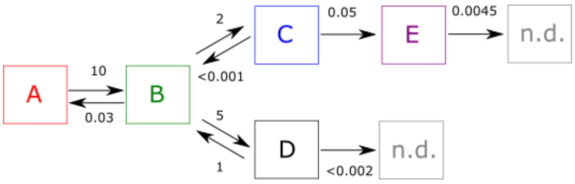
A
10
0.03
B
2
<0.001
C
0.05
E
0.0045
n.d.
5
1
D
<0.002
n.d.

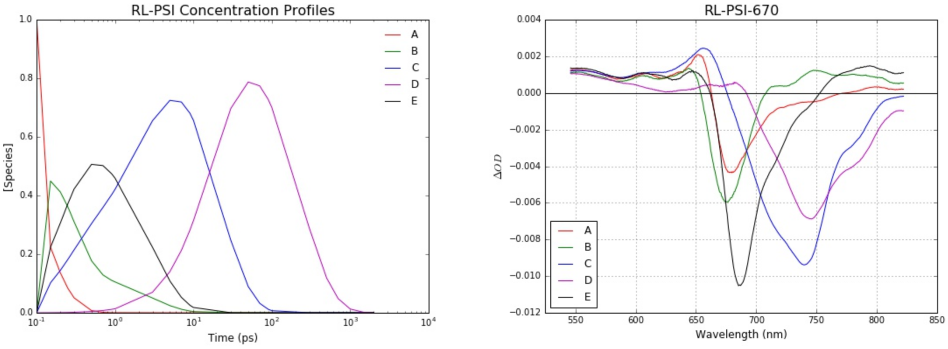
RL-PSI Concentration Profiles
[Species]
Time (ps)
A
B
C
D
E
RL-PSI-670
ΔOD
Wavelength (nm)

Pump energy and sample translation effects

Theoretical calculations

First we attempted theoretical estimation of the photons per particle measure using the model outlined by Müller et al. (1). Although the model relies heavily on knowing the value of the PSIcc extinction coefficient, a reasonable estimate can be obtained using known values for PSI RCs and the number of Chls present in the system. Thus the value of the photons per PSI particle is estimated to be around 1.1. This value indicates that double excitation of single PSI particle is possible and its effect on the resulting spectral kinetics should be investigated.

It should be noted that the photon density as measured in photon count per cm^2 in the measurements reported in this paper is less than 1.4 10^{14} cm^{-2}. This value is higher than approx. 0.14 10^{14} cm^{-2} in Müller et al. paper (2) but comparable to the one in Shleaev et al. paper - 10^{14} cm^{-2} (3) and significantly lower than 19 10^{14} cm^{-2} used by Donato et al. (4).

Recently Yoneda et al. (5) showed that in the case of PSII excitations at 10^{14} cm^{-2} level do not result in significant annihilation processes. While this is not directly applicable to the PSI due to roughly three times higher number of pigments per particle, it does suggest that in order to observe annihilation effects the excitation should exceed 10^{14} cm^{-2} level significantly.

Experimental tests

Additional tests were carried out to access whether the pump energy or the speed of sample translation can influence the results obtained in the measurements described in the main paper. Namely, we looked for the evidence of annihilation effects as well as the presence of “closed” PSI reaction centres.

A sample of FRL PSI was used in these measurements prepared under identical conditions as the one in the original measurements. The pump wavelength was chosen to be 670 nm because it corresponded to the highest sample absorption of all pump wavelengths, resulting in the highest number of absorbed photons. Thus this pump wavelength was most likely to show pump energy related effects. All other conditions were kept exactly the same as in the original measurements, except sample translation speed and pump energy.

The sample translation speed was varied between the stationary sample (indicated by ST=0 in Figs. below) and maximum translation rate allowable by our current setup (ST=10). Typically the measurements reported in the main text were carried out under ST=8-9 translation setting.

Pump energy was varied by introducing neutral density filter in to the pump beam. Three pump pulse energies were used compared to the original value (<10nJ, ND filter with OD=2) – ten times lower (ND filter with OD=3), two times lower (ND filter with OD=2.3) and ten times higher (ND filter with OD=1). Each time the attenuation was changed, the overlap of the pump and probe beams was optimized and time zero was determined.

Sample translation speed

For this test the pump energy was set at the same level as in the main text results. It should be noted that the number of scans recorded in these measurements was approximately 10 times smaller than in the main experiments thus resulting in noisier spectra. Furthermore, the data were collected just at four delay points: -100 ps, 0.5 ps, 3 ps and 2000 ps relative to the pump pulse.

As in Figure S1 the measurements performed at different sample translation speeds result in the spectra of identical shape. However there are some differences in the amplitude of the spectra. To assess these differences we have calculated the ratio of the bleach maximum at 2ns to the bleach maximum in 0.5 ps or 3 ps spectra. These results are shown in Figure S2.

There is a clear trend showing smaller ratios at slower speeds. The trend can be explained by the increasing proportions of the RCs being in the “closed” state. However at high translation speeds, there is little difference between ST=7 and ST=10 speed setting, indeed as smaller decrease occurs. This suggests that the effect of the “closed” RCs does influence the results at this translation speed.

Furthermore information about the presence of the “closed” RCs is contained in the -100 ps spectra (Figure S1, Bottom Right). However due low signal at this delay some smoothing had to be applied before analysis. Furthermore, in order to avoid baseline drift, a difference approach was used to evaluate the size of the bleach at 700 nm. Four reference points on both sides of the bleach maximum were chosen as reference. These points are indicated in the Figure S1, Bottom Right panel by open squares and circles. The size of the bleach was calculated as average of two differences – between bleach maximum and each reference point. The dependence of thus evaluated bleach size on sample translation speed is shown in Figure S3. This Figure also shows how the ratio of -100 ps bleach size to 2 ns bleach size changes with sample translation speed.

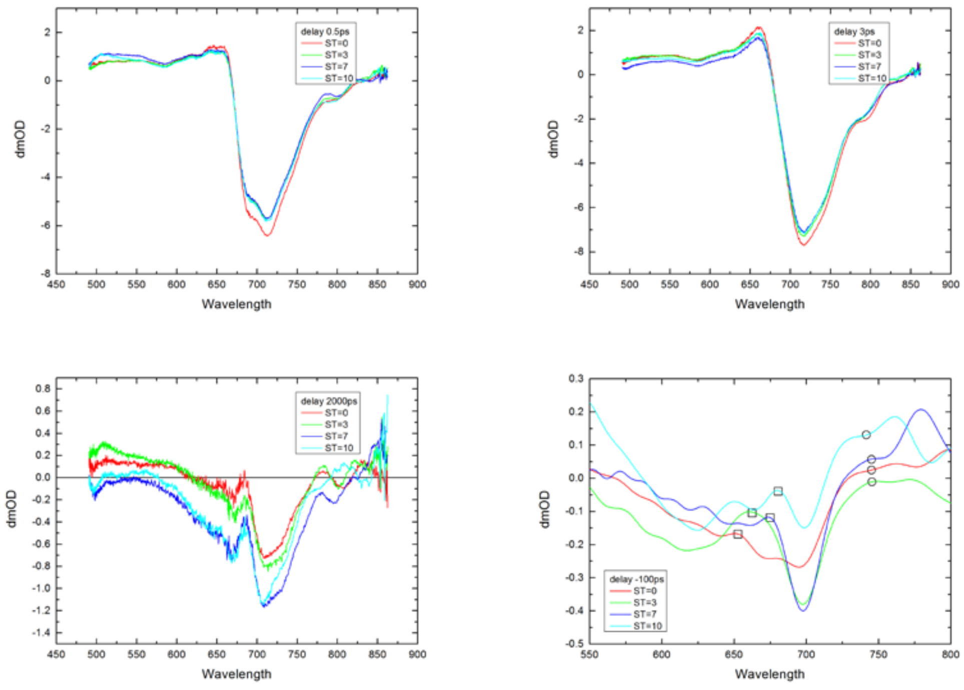

Figure S1 TA difference spectra of FRL PSI for four different sample translation speeds (ST=0,3,7,10) at four different delays: Top Left 0.5 ps, Top Right 3 ps, Bottom Left 2000ps, Bottom Right millisecond (-100 ps, probe before pump). The last spectrum has been smoothed using Loess algorithm and inverted. Open squares and circles indicate the reference levels used to evaluate the size of the bleach at 700 nm.

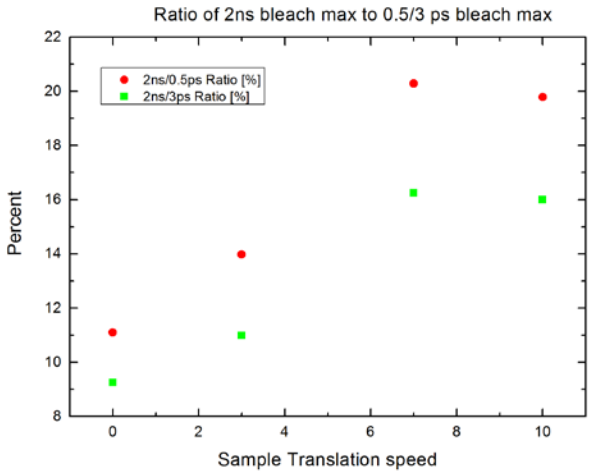

Figure S2 Comparison of the ratio between 2ns bleach and 0.5/3 ps bleach for different sample translaiton speeds

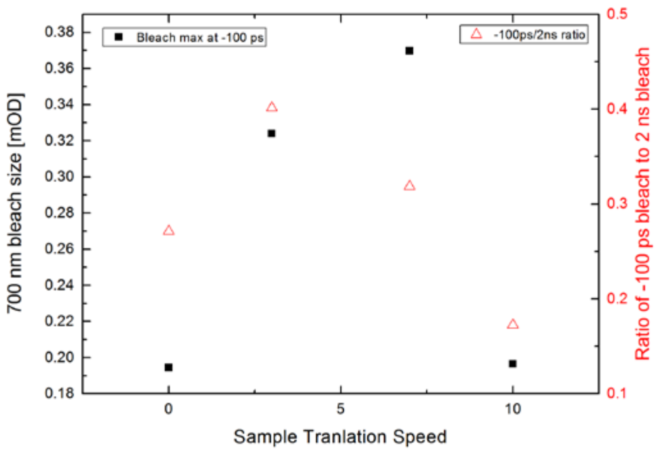

Figure S3 Analysis of the millisecond (-100 ps) spectrum. Black squares (Left scale) show dependence of the 700 nm bleach size on the sample translation speed. The size has been calculated using baseline reference points indicated in Figure above. Red triangles (Right scale) show ratio of millisecond (-100 ps) bleach size to the 2 ns bleach size.

Pump Energy

During these measurements, the sample was translated at the same speed as in the main text result case (ST=7-9) except for the case of the lowest pump energy where due to the low signal ST=1 setting had to be used. Moreover a higher number of scans was averaged in the case of the two lowest energies to produce acceptable signal to noise ratio.

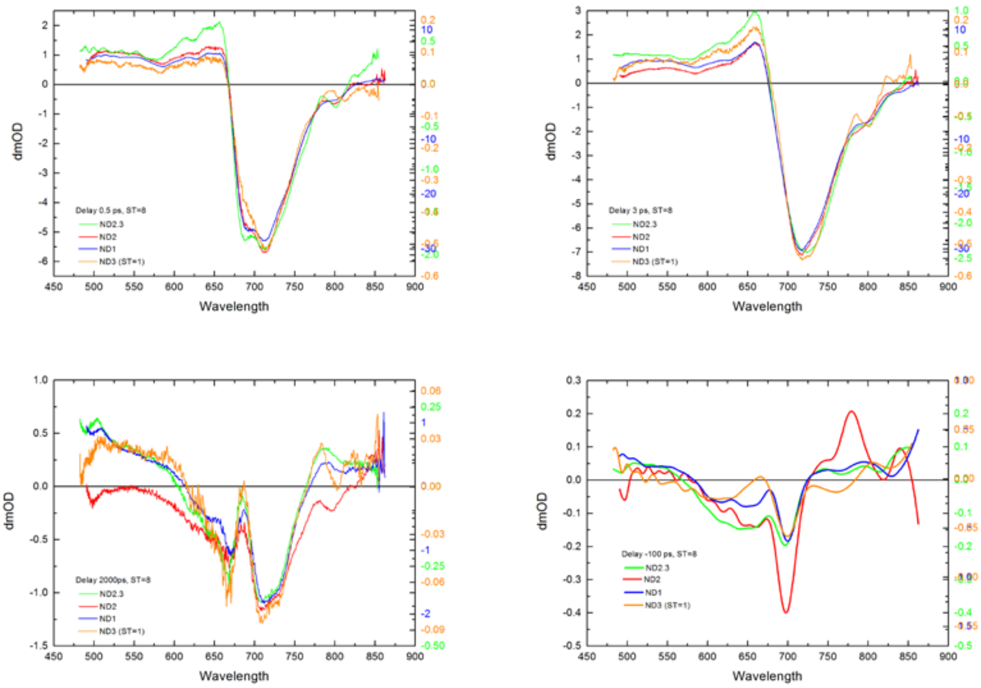

Figure S4 TA difference spectra of FRL PSI for four different pump levels (x0.1, x0.5, x1, x10 relative to the original measurements, indicated in the plot as ND3, ND2.3, ND2 and ND1 correspondingly) at four delays: Top Left 0.5 ps, Top Right 3 ps, Bottom Left 2000 ps, Bottom Right millisecond (-100 ps). The spectra in Bottom Right panel have been smoothed using Loess algorithm and inverted. All spectra are rescaled for comparison purposes.

Figure S4 shows the normalized spectra measured at the four pump energies and at four delay points. It is obvious that the shape of the spectrum stays the same for all pump levels. To assess the effect of the pump energy on the amplitude of the spectra we performed similar analysis as in translation speed case. Namely the ratio of 2 ns bleach maximum and 0.5/3 ps bleach maximum was calculated for all energy levels and is plotted in Figure S5.

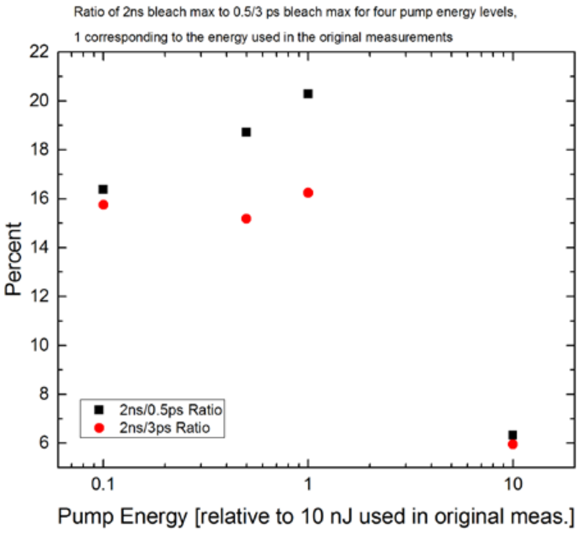

Figure S5 Comparison of the 2ns and 0.5/3 ps bleach ratio for the four pump energy levels.

With a 10-fold increase of pump-power it is clearly seen that this ratio reduces several-fold, indicative of exciton-exciton annihilation. For pulses up to 10-fold weaker we observed a weak trend that reduces the ratio for 2ns/0.5ps bleach amplitudes, but not for the 2ns/3ps (Figure S5). Determination of the 500 fs bleach amplitude may be affected more strongly by the coherent artefact at higher pump powers, therefore considering the 3ps amplitude, we conclude that loss of excited state population through exciton-exciton annihilation is minimal under our experimental conditions.

In conclusion, the experimental tests suggest that the annihilation and “closed” PSI RCs even if present under typical experimental condition used in this work, do not influence the processes of energy and charge transfer and relative yield for radical pair formation substantially.

Estimation of Charge Separation Efficiency

We also attempted to compare the charge separation efficiency between WL and FRL PSI samples. Namely the ratio of the bleach integrals at 2ns and 0.3 ps was used as a measure representative of charge separation efficiency. Figure 6 shows region of the spectra that was used for this estimation.

Surprisingly 670 nm pump data show that the ratio is higher in FRL case – 0.3174 compared to 0.2829 in WL case. The same is true for 700 nm pump – 0.3308 vs. 0.2156.

Although these calculations seem to suggest that CS in FRL PSI is more efficient, in our opinion this type of measure is not reliable enough to make strong conclusions. For example, contribution of still present radiative decay could affect the obtained numbers substantially.

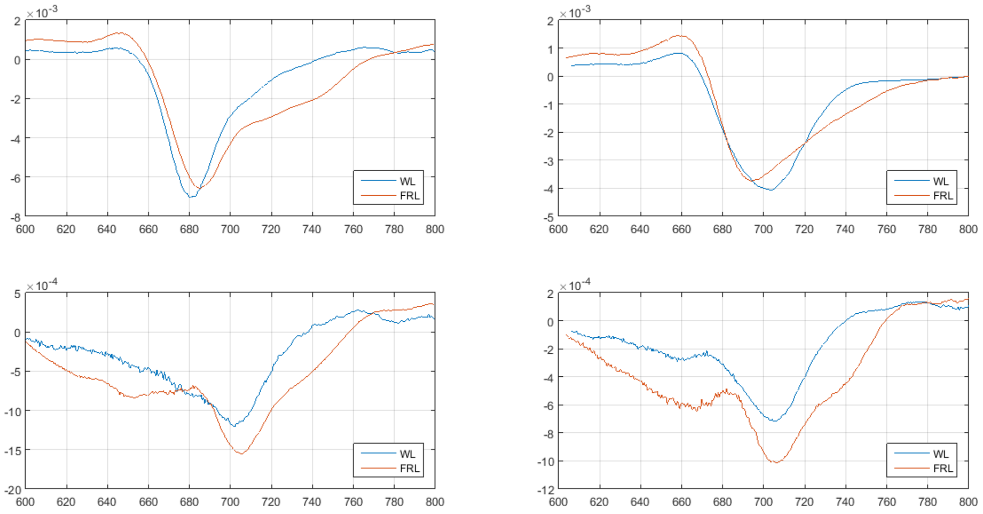

Figure S6 Experimental spectra used in CS efficiency estimation. Left panel 670 nm pump, Right panel 700 nm pump. Top panels show 0.3 ps spectra and Bottom panels 2 ns spectra

References

1. Müller, M.G., M. Hucke, M. Reus, and A.R. Holzwarth. 1996. Annihilation Processes in the Isolated D1-D2-cyt-b559 Reaction Center Complex of Photosystem II. An Intensity-Dependence Study of Femtosecond Transient Absorption † , ‡. J. Phys. Chem. 100: 9537–9544.

2. Müller, M.G., J. Niklas, W. Lubitz, and A.R. Holzwarth. 2003. Ultrafast transient absorption studies on Photosystem I reaction centers from Chlamydomonas reinhardtii. 1. A new interpretation of the energy trapping and early electron transfer steps in Photosystem I. Biophys. J. 85: 3899–922.

3. Shelaev, I. V., F.E. Gostev, M.D. Mamedov, O.M. Sarkisov, V.A. Nadtochenko, V.A. Shuvalov, and A.Y. Semenov. 2010. Femtosecond primary charge separation in Synechocystis sp. PCC 6803 photosystem I. Biochim. Biophys. Acta - Bioenerg. 1797: 1410–1420.

4. Donato, M. Di, A.D. Stahl, I.H.M. Van Stokkum, R. Van Grondelle, and M.L. Groot. 2011. Cofactors involved in light-driven charge separation in photosystem i identified by subpicosecond infrared spectroscopy. Biochemistry. 50: 480–490.

5. Yoneda, Y., T. Katayama, Y. Nagasawa, H. Miyasaka, and Y. Umena. 2016. Dynamics of Excitation Energy Transfer Between the Subunits of Photosystem II Dimer. J. Am. Chem. Soc. : jacs.6b04316.
