# Supplementary material for: Femtosecond Visible Transient Absorption Spectroscopy of Chlorophyll f-Containing Photosystem I
Source: Biophys J. 2017 Jan 24;112(2):234–49. doi: 10.1016/j.bpj.2016.12.022 (PMC5266252; doi:10.1016/j.bpj.2016.12.022)
Supplement: Document S2. Article plus Supporting Material [file mmc2.pdf]

Biophysical Journal
Article

Femtosecond Visible Transient Absorption Spectroscopy of Chlorophyll *f*-Containing Photosystem I

Marius Kaucikas,[1] Dennis Nürnberg,[1] Gabriel Dorlhiac,[1] A. William Rutherford,[1] and Jasper J. van Thor[1,*]
[1]Department of Life Sciences, Imperial College London, London, United Kingdom

ABSTRACT Photosystem I (PSI) from *Chroococcidiopsis thermalis* PCC 7203 grown under far-red light (FRL; >725 nm) contains both chlorophyll *a* and a small proportion of chlorophyll *f*. Here, we investigated excitation energy transfer and charge separation using this FRL-grown form of PSI (FRL-PSI). We compared femtosecond transient visible absorption changes of normal, white-light (WL)-grown PSI (WL-PSI) with those of FRL-PSI using excitation at 670 nm, 700 nm, and (in the case of FRL-PSI) 740 nm. The possibility that chlorophyll *f* participates in energy transfer or charge separation is discussed on the basis of spectral assignments. With selective pumping of chlorophyll *f* at 740 nm, we observe a final ~150 ps decay assigned to trapping by charge separation, and the amplitude of the resulting $P700^{+\bullet}A_1^{-\bullet}$ charge-separated state indicates that the yield is directly comparable to that of WL-PSI. The kinetics shows a rapid 2 ps time constant for almost complete transfer to chlorophyll *f* if chlorophyll *a* is pumped with a wavelength of 670 nm or 700 nm. Although the physical role of chlorophyll *f* is best supported as a low-energy radiative trap, the physical location should be close to or potentially within the charge-separating pigments to allow efficient transfer for charge separation on the 150 ps timescale. Target models can be developed that include a branching in the formation of the charge separation for either WL-PSI or FRL-PSI.

INTRODUCTION

Photosystem I (PSI) uses light to drive the oxidation of plastocyanin or cytochrome *c* and the reduction of ferredoxin as a part of oxygenic photosynthesis. It is a large multisubunit transmembrane protein, the structure of which has been determined with 2.5 Å resolution from the cyanobacterium *Thermosynechococcus elongatus* (1). The main cofactor-containing part of the PSI reaction center (RC) is made up of two nearly symmetrical membrane-spanning subunits, PsaA and PsaB, which contain 96 chlorophyll (Chl) molecules. Only six of these are considered to be involved in charge separation and stabilization. These six Chls form part of two symmetrical electron transfer pathways, each made up of three Chls *a* (P, A, and A_0) and a phylloquinone (A_1), with a single iron sulfur center, F_X, acting as the electron acceptor from the quinones of both branches. At the luminal side of PSI, two P Chls, one from each branch, are in close proximity and have partial overlap of their aromatic rings, which leads to enhanced electronic coupling, red-shifting the absorption maximum to 700 nm. This pair of Chls is usually denoted P700 (2,3). Charge separation takes place in both branches (4,5).

There has been some debate about the sequence of events that occur upon excitation and charge separation in PSI. For many years, it was thought that the Chl pair P700 was the primary electron donor, i.e., the species that bore the first excited singlet state immediately before charge separation. However, evidence indicating that the monomeric Chl, A, is the primary donor has been presented.

The assignment of the primary electron acceptor is conditioned by the identity of the primary donor. If P700 is the primary donor, then the adjacent pigment, A, is the likely candidate for the primary acceptor, with $P700^{+\bullet}A^{-\bullet}$ as the first radical pair (RP1). In contrast, in the model where A is the primary donor, then A_0 would be the primary acceptor, with $A^{+\bullet}$ $A_0^{-\bullet}$ as RP1. In both of these models, the second radical pair, RP2, is in most cases considered to be $P700^{+\bullet}A_0^{-\bullet}$. The third radical pair, RP3, is $P700^{+\bullet}A_1^{-\bullet}$, which is stable in the time range of nanoseconds to tens of nanoseconds, with electron transfer toward F_x occurring in 200 ns and 20 ns on the PsaA branch and PsaB branch, respectively (2,6).

The identity of the primary electron donor, and thus the sequence of radical pairs produced in PSI, remains

Submitted August 25, 2016, and accepted for publication December 1, 2016.
*Correspondence: j.vanthor@imperial.ac.uk
Editor: Elsa Yan.
http://dx.doi.org/10.1016/j.bpj.2016.12.022
© 2017 Biophysical Society.
This is an open access article under the CC BY license (http://creativecommons.org/licenses/by/4.0/).

uncertain, with recent work reporting evidence that $P700^{+\bullet}$ is formed at the earliest times. This brings into question the model in which the monomeric Chl, A, is the primary donor.

Light harvesting within PSI is extensively described in the literature (see reviews in (2,3,7)). However, there are still open questions about the initial steps that take place after absorption of a photon and the processes that occur before charge separation.

Spectroscopic studies of different PSI samples have indicated the presence of antenna Chls that have absorption maximum at wavelengths above 700 nm. The exact absorption wavelengths and numbers of these so-called red Chls are species dependent (8–11). The role of red Chls in energy and charge transfer has been investigated for PSI from different cyanobacteria (see Ref. (8) and references therein); however, the precise role of red-shifted Chls in PSI is still being discussed. The Chl *a* pigments in dimeric or oligomeric forms have been suggested to be those corresponding to the red Chls (12). Furthermore, charge separation with 808 nm excitation has been demonstrated even at room temperature (13). Schlodder et al. (13) suggested that direct excitation of charge-transfer states resulting from the ring overlap between the two Chls within P700 in the RC is responsible for this. In an earlier study, Schlodder et al. (12) demonstrated that the fluorescence of red pigments was sensitive to the redox state of P700, and presented an analysis of the possible Chl aggregates from the crystallographic coordinates. In this study, we consider the role of red Chl *f* pigments that result from adaptation of the organism to a far-red light (FRL) environment, and their possible role in light harvesting and charge separation.

Chl *f* was discovered recently in cyanobacteria (14) and was found to have the most red-shifted Qy band of all forms of Chls. Niedzwiedzki et al. (15) examined the properties of the Chl *f* excited state in organic solvents and concluded that the singlet excited-state lifetime of Chl *f* is 5.6 ns at room temperature. They expressed doubt that an efficient Chl *f*-to-Chl *a* energy transfer would be possible. These conclusions were based on the relatively low-fluorescence quantum yield of 0.16 and the necessity for a significant uphill energy transfer from Chl *f* (706 nm absorption λ_{max} in Pyr) to Chl *a* (671 nm absorption λ_{max} in Pyr) to occur. However, although the ~500 cm^{-1} (62 meV) uphill energy gap is feasible at room temperature, given kT (25.7 meV) and the overlap of the broad absorption spectra of the Chls, the energy transfer will occur only at a reduced rate. The reduced quantum yield does not affect transfer on the ~100 ps timescale seen for the Chl *f* decay in this study, and the effective uphill rate will depend on the Franck-Condon term in the Marcus equation and the temperature.

The presence of Chl *f* in PSI is expected to have an important effect on excitation transfer, if it plays a light-harvesting role as proposed in the literature (16,17). It has been suggested that Chl *f* does not play a role in charge separation within the RC, but little or no experimental evidence exists relevant to this issue. Here, we applied ultrafast time-resolved absorption spectroscopy to Chl *f*-containing PSI to test this proposition.

Ultrafast time-resolved studies of PSI

Numerous ultrafast transient absorption (TA) and fluorescence studies of PSI have been presented, but no consensus regarding the model of energy and charge transfer in PSI after absorption of a photon has been reached. Most of the proposed models fall into one of two categories: 1) transfer to trap limited and 2) trap limited. Transfer-to-trap-limited models suggest a slow (typically ~20 ps) excitation transfer time from the antenna to the charge-separating Chls, whereas the charge-separation step is assumed to take 1–10 ps (18–21). In contrast, the trap-limited model proposes that energy equilibration between the antenna and the charge-separating Chls occurs within 3 ps, whereas charge separation and formation of the first radical pair might take ~20 ps (22–24). Below, we briefly summarize some of the recent publications in this field.

Müller et al. (25) used the green alga *Chlamydomonas reinhardtii* PSI for TA experiments because it did not contain red Chls, thus allowing fast excitation and charge-transfer steps to be resolved. The model used for data fitting included subpicosecond equilibration within the excited antenna and the charge-separating Chls, and a 2 ps time constant for the excitation transfer between the two types of pigments. Central to this model was that the charge separation occurred with a 6–9 ps time constant. The formation of the second and third radical pairs occurred with 15–20 ps and 35–40 ps time constants, respectively.

The trapping kinetics of *T. elongatus* PSI, which contains red Chls, was studied by Slavov et al. (8) using time-resolved fluorescence. They demonstrated that even in the presence of red Chls, the kinetics follows a trap-limited scheme for both PSI trimers and monomers. However, the rate of trapping was slower compared with *C. reinhardtii* PSI (25). The dominant equilibration time found for PSI containing red Chls was 5–7 ps, with some additional slower phases of 25–44 ps (8).

Using time-resolved fluorescence emission measurements, van Stokkum et al. (26) demonstrated that *Synechococcus* sp. WH 7803 PSI is free of red Chls. Their results obtained at room temperature showed two decay components with lifetimes of 7.5 ps and 18 ps. Target fitting of the data assigned the first lifetime to equilibration between one pool of antenna Chls (Ant2)* and a combined excited state of the charge-separating Chls and another antenna Chl (Ant1/RC)*. The second lifetime was assigned to formation of the radical pair $P700^{+\bullet}A_0^{-\bullet}$. The authors also showed that a similar model could be used to describe the time evolution of the fluorescence emission time for other cyanobacteria PSI even in the presence of red Chls. It is worth noting that van Stokkum et al. discussed published evidence that *Chlamydomonas*

PSI as used by Müller et al. (25) might contain red Chls emitting 715 nm at 77 K (26).

Shelaev et al. (27) performed TA measurements on PSI from *Synechocystis* sp. PCC 6803 with 20 fs temporal resolution, and proposed that 1) charge separation occurs in <100 fs; 2) energy transfer from the antenna to the RC occurs in 5 ps; and 3) formation of the secondary radical pair takes 25 ps. However, they did not consider the presence of the red Chls in PSI of this species. Previous reports using the PSI from the same species (*Synechocystis* 6803) (e.g., (23,26)) suggested the presence of red Chls. Whereas Melkozernov et al. (23) considered the possibility of a red Chl contribution to a ~100 ps 705 nm bleach in *Synechocystis* PSI, Shelaev et al. (27) assigned the band at 705 nm to $P700^{+\bullet}$. The latter group went on to investigate the effect of the excitation wavelength on primary photochemistry in PSI (28) and showed that excitation with 670 nm or 700 nm led to large absorption changes in the antenna Chls and annihilation effects that resulted in anomalous kinetics, and spectra that obscured the kinetics of the charge separation. They suggested that earlier studies had been dominated by such effects, and found that these problems were reduced when weaker excitation was given by using longer-wavelength excitation that had only a partial overlap with the Chl absorption bands.

Recently, Kompanets et al. (29) performed TA measurements of *Arthrospira platensis* PSI, which contains extremely red-shifted Chls. They observed two bleaches developing at 710 nm and 730 nm with 1 ps and 5.5 ps time constants, respectively. The authors concluded that the red Chls were populated by excitation transfer from bulk antenna Chls.

Visible pump/infrared probe TA spectroscopy of *Synechococcus elongatus* PSI was performed by Di Donato et al. (9). The infrared measurements clearly separated excited-state decay from charge-separation processes spectrally. The results showed a heterogeneous excited-state decay, and the charge separation and branching were modeled either with inclusion of a red Chl *a* pool or with separate excited antenna pools. One model proposed by the authors includes a red Chl compartment connected to the bulk antenna only, and assumes that the red Chls and P700 are not strongly connected and the energy transfer kinetics between them takes a few picoseconds. Moreover, they concluded that once the excitation reaches the RC, the charge separation occurs on a subpicosecond timescale. They suggested that charge separation starts with the monomer Chl A, not P700, and thus the initial radical pair consists of $A^{+\bullet}$ and $A_0{}^{-\bullet}$. Phylloquinone A_1 is reduced with a time constant of 6 ps and the final relaxed radical pair $P700^{+\bullet}A_1{}^{-\bullet}$ is formed by 40 ps. A similar model was proposed previously by Müller et al. (6) and Holzwarth et al. (30) on the basis of modified kinetics in site-directed mutations positioned near A_0.

Chauvet et al. (31) were able to resolve the $A_0{}^{-\bullet}/A_0$ spectrum spectrally without chemical reducing agents. They used a mutant of *Synechocystis* sp. PCC 6803 in which electron transfer from the A_0 state to A_1 was slowed down (from 30 ps to several nanoseconds), leaving a long-lived $A_0{}^{-\bullet}$ state. The resulting $A_0{}^{-\bullet}/A_0$ spectrum shows a bleach maximum at 684 nm as well as a minor positive signal in the 640–660 nm and 700–740 nm regions (see Fig. 7 *b* in Ref. (31)). The availability of this spectrum enables better analysis and interpretation of PSI TA results.

A simplified summary of models for the energy- and charge-transfer steps suggested in the above-cited works is presented in Fig. 1. Typical transition times between different steps are also indicated. As can be seen from Fig. 1, there are still many fundamental differences between the different models, but some general trends can be observed. The typical equilibration time between the antenna and the charge-separating Chls at the center of the RC is often reported to be on the order of 2–8 ps. According

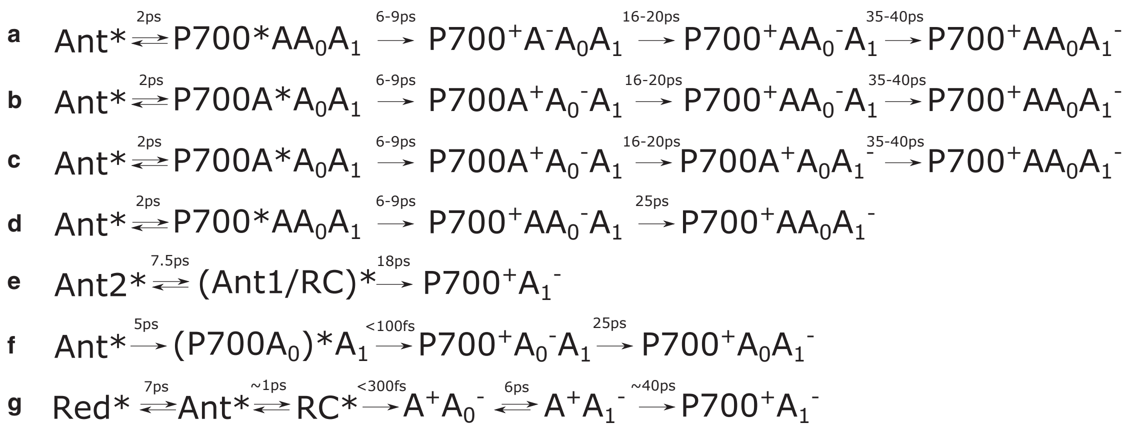

FIGURE 1 (*a–g*) Simplified PSI charge-separation schemes suggested by different authors: (*a–d*) Müller et al. (25,30), (*e*) van Stokkum et al. (26), (*f*) Shelaev et al. (27) and (*g*) Di Donato et al. (9). To simplify comparison, the indicated time constants are typical values observed by the authors of the corresponding studies and do not represent exact rates. Ant, antenna Chls *a*; P700, Chl *a* pair; A and A_0, Chl *a* pigments; A_1, phylloquinone; RC, reaction center; red, red Chl *a* pool.

to all of the models discussed here, a secondary charge-transfer step happens with a 20–40 ps time constant. The most significant difference between the results behind these models is the rate at which charge separation occurs. Typical transition times from the excited RC state to the primary charge-separation state are given with 6–18 ps time constants according to the models in Fig. 1, *a–e*. The models in Fig. 1, *f* and *g*, suggest that the primary charge separation occurs on a subpicosecond timescale.

Note that the models summarized in Fig. 1 used different experimental techniques, were conducted under different optical conditions, and used samples of various origins. The presence of FRL-absorbing Chl *f* in the PSI core would allow better differentiation between energy-transfer and charge-separation processes using visible detection.

In this work, we performed visible TA spectroscopy of *C. thermalis* PCC 7203 PSI samples, including both white light (WL)-grown PSI (where all Chls are Chl *a*) and FRL-grown PSI containing Chl *f*. Under conditions of pumping in the visible spectral range that are selective for either blue-shifted Chl *a* antenna pigments, red-shifted Chl *f* pigments, or pigments that favor direct charge separation, we compared TA measurements for energy-transfer and charge-separation processes.

MATERIALS AND METHODS

Sample

Cells of *C. thermalis* PCC 7203 were grown in liquid BG11 medium (32) at 30°C under FRL (750 nm; Epitex (Ushio Epitex, Kyoto, Japan)) of 45 μE m^{-2} s^{-1} (optical power meter; Newport, Irvine, CA)) and WL of 30 μE m^{-2} s^{-1} (Quantitherm light meter; Hansatech, King's Lynn, UK). PSI solubilized by *n*-dodecyl-β-maltoside (β-DDM) was purified by sucrose density gradient centrifugation. The samples were in a buffer of 50 mM MES-NaOH, 5 mM $CaCl_2$, 10 mM $MgCl_2$, and 0.04% (w/v) β-DDM (pH 6.5) with 40 mM sodium ascorbate and 50 μM phenazine methosulfate as redox mediator. The samples were transferred to a cell (Harrick Scientific, Pleasantville, NY) with 1 mm CaF_2 windows and a 25 μm spacer. Purity was assessed by absorption and fluorescence measurements. The absorption spectra for both WL- and FRL-PSI samples are presented in Fig. 2. The optical density at 675 nm was typically 0.6–0.7.

Setup

The setup for visible TA measurements was described previously (33). Briefly, the 800 nm output from a Ti-Sapphire laser (90 fs, 1 kHz, 0.85 mW; Hurricane, Spectra Physics (Newport Spectra-Physics, Santa Clara, CA)) was divided between an optical parametric amplifier (OPA-800C, Spectra Physics) and a WL generator. The amplifier produced pump radiation tunable in the 600–800 nm region. We used three pump wavelengths in the experiments: 670 nm, 700 nm, and 740 nm. The typical bandwidth of the pump radiation was ~20 nm and the energy reaching the sample was <10 nJ. Given a beam diameter of 0.175 mm at the sample position, this energy corresponds to 0.04 W/cm^2 or 42 μJ/cm^2.

The WL was generated using a sapphire plate and spanned the spectral region from 400 to 800 nm. The delay between pump and probe pulses was varied from −100 ps to 2 ns using a retroreflector mounted on a delay line (M-IMS400CCHA, Newport) introduced into the pump beam path. The −100 ps (probe − pump) measurement represents the TA at 1 ms with inverted amplitude, and is subtracted as a background from the positive delay points.

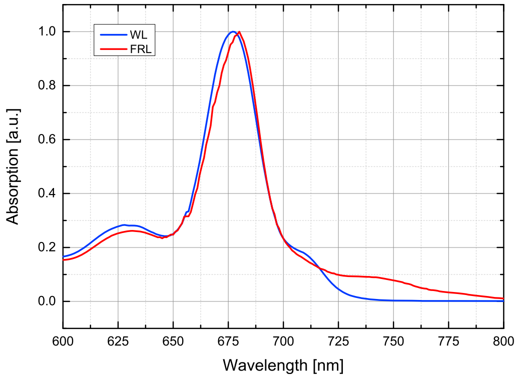

FIGURE 2 Absorption spectra of WL- and FRL-PSI. The spectra were normalized with respect to the 675 nm maxima. Evidence for the presence of FRL-absorbing Chl *f* is seen in the absorption extending beyond 750 nm in the FRL-PSI sample. To see this figure in color, go online.

The sample was continuously moved in a Lissajous pattern using an in-house-built sample translator to minimize repeated exposure of the same sample volume.

After it passed through a sample, the probe radiation was analyzed using an in-house-built prism spectrometer employing a 1024 pixel CCD camera. Data collection and preprocessing were performed with the use of custom software written using LabVIEW (National Instruments, Austin, TX).

All measurements were performed at room temperature.

Data analysis

The data were analyzed using global analysis and target analysis (34), as well as lifetime-density (35) methods. Specifically, the freely available Glotaran (36) and Global Analysis Toolbox (37) packages were employed for this purpose, which included modeling of the instrument response function and chirp correction.

RESULTS

WL-grown PSI

Transient absorption measurements on the WL-PSI sample were performed at two pump wavelengths: 670 nm and 700 nm. With 670 nm excitation, preferential excitation of the antenna Chls is expected, whereas 700 nm excitation is expected to result in more selective excitation of the charge-separating pigments in the RC (25). Difference spectra at selected delays are presented in Fig. 3.

It should be noted that the −100 ps delay spectrum has been subtracted from each spectrum in Fig. 3. The −100 ps spectra represent the long-lived component of the spectral evolution present at 1 ms (22,25) and are presented in Fig. 4. The subtraction of −100 ps spectra from the data allowed us to focus on the changes in the spectra that occurred in the first 2 ns after excitation. With the applied raster scanning speeds, the average overlap with the pumped volume of the previous pump-probe measurement was ~0.2, which minimized the contribution to the ultrafast pump-probe amplitudes while still allowing measurement of the

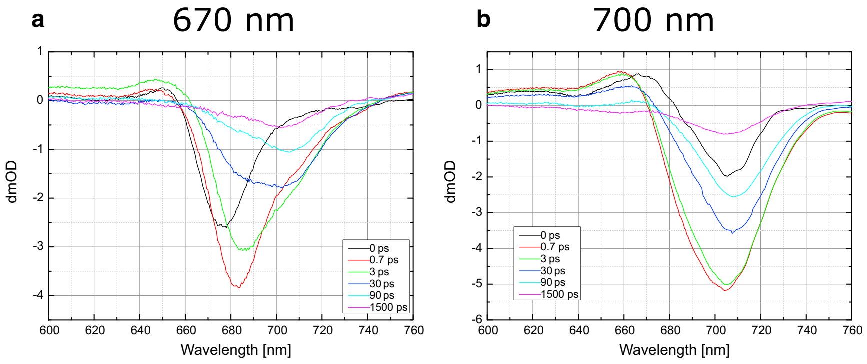

FIGURE 3 (*a* and *b*) TA spectra at selected delay points (in picoseconds) for WL-PSI with pump wavelengths of (*a*) 670 nm and (*b*) 700 nm. To see this figure in color, go online.

millisecond spectrum (Figs. S1–S3 in the Supporting Material).

In line with comparable measurements of Chl *a*-containing PSI reported previously (25), there is a significant difference between the data obtained with excitation at 670 nm and those obtained at 700 nm. The 670 nm pump excitation is absorbed mostly in the antennae, resulting in a 675 nm photobleaching (PB) band at time zero. Within the first picosecond, a new PB band at 682 nm develops with a larger amplitude than the 675 nm bleach at 0 ps. It is important to note that in the 0.7 ps spectrum, there is already a shoulder starting to develop at 706 nm. A spectral broadening also occurs on the subpicosecond timescale, which is generally assigned to exciton equilibration processes (25). The bleach maximum moves to 686 nm in the 3 ps spectrum, with its amplitude decaying compared with the 0.7 ps spectrum. The 10 ps and subsequent spectra clearly show dual bleach shapes: one centered at 688 nm and the other centered at 706 nm.

With 700 nm excitation, the initial bleach observed at 0 ps has a minimum at 704 nm that rapidly moves to 696 nm by 0.1 ps. This uphill movement of excitation was also observed previously for *Chlamydomonas* PSI (22,25) and *Synechocystis* 6803 PSI (23). At 0.7 ps the bleach spectrally broadens, which in analogy to the previous *Chlamydomonas* and *Synechocystis* PSI studies (see Refs. 22,23,25) may be dominated by the exciton equilibration in the RC pigments primarily, and has a minimum at 704 nm. These dynamics will additionally have contributions from exciton relaxation occurring in antennae pigments, but quantification is not feasible in the absence of either structure-based theory or experiment with regard to the selectivity of excitation at 700 nm. The subsequent spectra show a decay of this bleach together with a small shift of its minimum to 706 nm (Fig. 3). The signal at 706 nm is distinctive for TA of the WL-PSI, and its origin, assigned to Chl *a*, is discussed further below in light of previous studies of red pigments in PSI (see Discussion).

The 670 nm excitation data obtained with up to 100 ps delays are mostly similar to those obtained by Müller et al. (25) in *Chlamydomonas* PSI. However, there is one significant difference: the emergence of the 706 nm bleach in the current data (see Fig. 3 spectra obtained at 0.7 ps and later). The 706 nm bleach becomes a dominant feature in the ≥ 1 ns delay spectra. Fig. 5 shows a comparison of the 1.5 ns spectra for 670 nm and 700 nm excitation wavelengths. The origin and assignment of the 706 nm bleach are addressed in more detail in the Discussion section below.

The temporal development of the signal can be visualized using a lifetime-density map (25,30). In contrast to target analysis, the lifetime-density map is not model dependent and can highlight the distributions of lifetimes. A lifetime map for WL-PSI is presented in Fig. 6. For 670 nm excitation, the most notable lifetimes longer than the subpicosecond signals are 2 ps (with a negative amplitude) and ~40 ps in the 680–690 nm region, and 6 ps (positive amplitude) in the

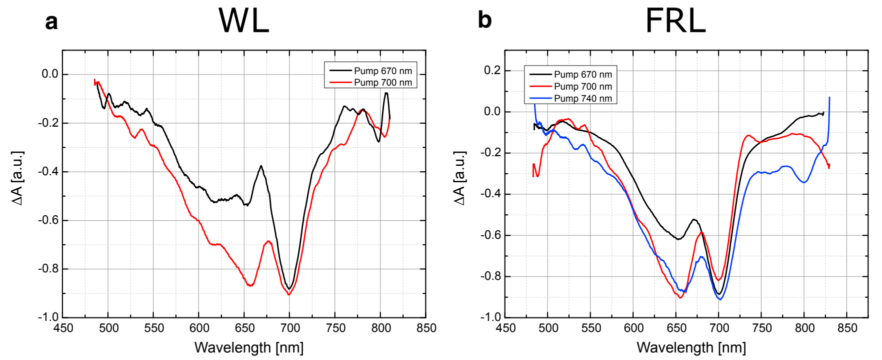

FIGURE 4 (*a* and *b*) Millisecond spectra of WL-PSI (*a*) and FRL-PSI (*b*) with different pump wavelengths. The spectra have been inverted because at negative delays, the probe and the pump pulses effectively swap places. To see this figure in color, go online.

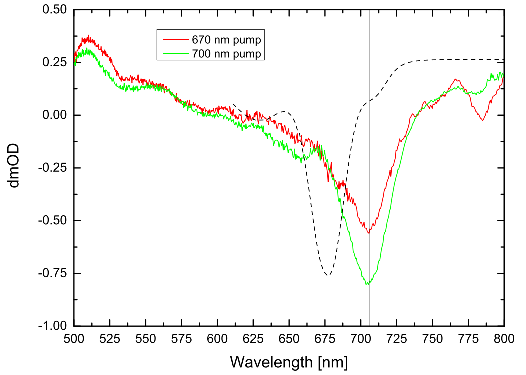

FIGURE 5 Comparison of 1.5 ns spectra for pump wavelengths of 670 nm and 700 nm. An inverted absorption spectrum of the WL-PSI sample is shown by the black dashed line. To see this figure in color, go online.

706 nm spectral region. With 700 nm excitation, the map is more complicated, with the two most prominent features being a 40 ps broad peak with negative amplitude and a 1500 ps peak with a negative amplitude in the 710 nm region.

FRL-grown PSI

We used three pump wavelengths for FRL-PSI: 670 nm, 700 nm, and 740 nm. The 740 nm pump wavelength should preferentially excite Chl *f* present in the sample. Difference spectra at selected delays are presented in Fig. 7. The −100 ps (millisecond) spectra have been subtracted and are presented in Fig. 4. Investigation of the power-density dependence and calculation indicated excitation levels of ~1 per complex. Measurements of the relative charge-separation efficiency at a 10-fold reduced power density were not significantly changed, indicating that the exciton-exciton annihilation contribution was minimal (Supporting Materials and Methods; Figs. S4 and S5). Thus, under the condition of a minimized background of closed PSI at ~20% and in the absence of detectable exciton-exciton annihilation, our investigation of relative charge-separation efficiency and energy-transfer kinetics is dominated by one-photon processes and an open PSI, and avoids an interpretation of small-amplitude differences.

For the 670 nm excitation, which is considered to be specific for Chl *a* pigments, the initial time zero bleach occurs at 685 nm; however, by 0.7 ps the bleach position had already moved to 690 nm (Fig. 7). Furthermore, starting with 0.7 ps, a broad shoulder on the red side of the bleach (710–760 nm) starts to develop, indicating a rapid transfer to Chl *f* pigments. The 3 ps difference spectrum shows a multimodal character with the main bleach at 740 nm. Other minima in the 3 ps spectrum are at 690 nm, 710 nm, and 780 nm. All spectra obtained later than 10 ps are dominated by the main 740 nm bleach, with the minimum moving further toward red, from 740 to 745 nm.

The TA measurements obtained with 700 nm pump excitation show a spectral evolution that is similar to the data obtained with 670 nm pump excitation. The initial bleach at 690 nm migrates toward the far-red wavelengths (710–760 nm), and by 10 ps the main dominating bleach is at 740 nm. It is noted that the 3 ps spectrum of the 670 nm excitation data shows a multimodal character with additional bleaches at 710 nm and 780 nm. The spectral evolution after 10 ps follows the same pattern as the measurements with the 670 nm pump excitation.

Pumping Chl *f* directly at 740 nm shows spectral kinetics consistent with the later stages (>20 ps) of the previous two pump wavelengths. The bleach minimum at 0.7 ps is seen at 740 nm with a small shoulder at 780 nm. Subsequent spectra obtained later than 0.7 ps do not show major changes in the shape of the spectrum, apart from a small drift of the minimum from 740 nm toward a further red wavelength at 745 nm.

The 1.5 ns delay FRL-PSI spectra are very similar to those observed for WL-PSI and additionally are independent of the wavelength of the initial excitation (Figs. 5 and 8). A single bleach centered at 706 nm dominates the spectra for delays longer than 1 ns, as shown in Fig. 8. This is taken as an indication that the same Chls responsible for the 706 nm bleach in the WL-PSI are present in the FRL-PSI.

Lifetime-density maps were used to visualize the time evolution of the spectra and are presented in Fig. 9. Compared with those obtained for WL-PSI, the maps for

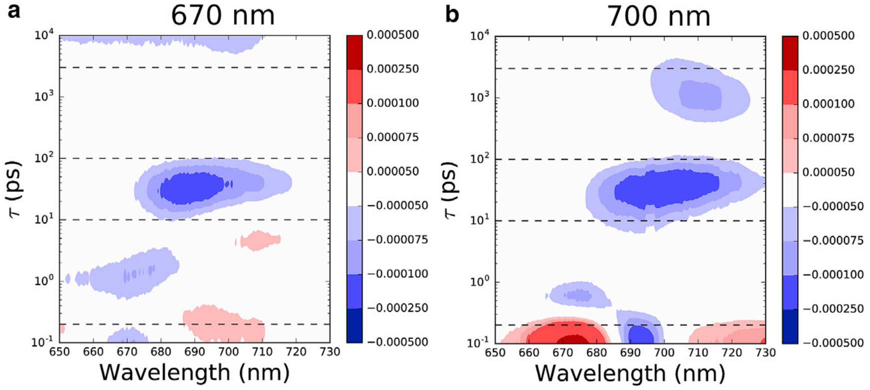

FIGURE 6 Lifetime-density map for WL-PSI data: (*a*) 670 nm pump and (*b*) 700 nm pump. Dashed lines indicate lifetimes observed in sequential model fits (see "Fit results" section). To see this figure in color, go online.

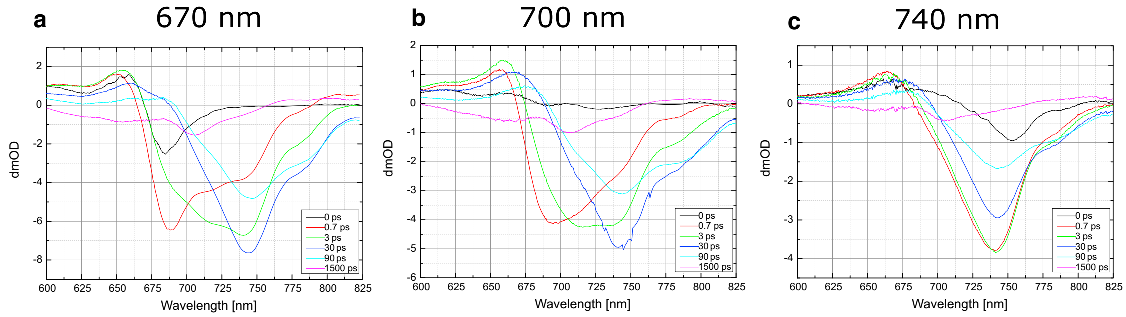

FIGURE 7 (*a–c*) TA spectra at selected delay points for FRL-PSI with pump wavelengths of (*a*) 670 nm, (*b*) 700 nm, and (*c*) 740 nm. Delay times are given in picoseconds. To see this figure in color, go online.

670 nm and 700 nm pump excitation are much more complex. Apart from subpicosecond components, the most notable lifetimes in the 740 nm region are 2–6 ps (positive amplitude) and ~100 ps (negative amplitude). The 690 nm region shows a broad range of lifetimes having negative amplitude stretching from subpicoseconds to 30 ps. Furthermore, a 200 ps (positive amplitude) lifetime is observed in this region as well. The most prominent feature in the lifetime map for 740 nm pump excitation is a negative amplitude for the 100 ps lifetime in the 740 nm spectral region.

Fit results

Fitting the sequential model

WL sequential fitting results. The results of fitting a homogeneous, or fully sequential, model to the WL-PSI data are presented in Fig. 10. Four sequential stages were used for the measurements made with 670 nm pump excitation. The first compartment (compartment A), with a lifetime of <200 fs, represents exciton equilibration between antenna Chls. The second compartment (compartment B), with a bleach maximum at 685 nm, decays with 1.5 ps time constant.

This time constant agrees well with that observed for the transfer from the antenna to the RC in some of the models shown in Fig. 1. Thus, compartment B represents the excited state of the antenna Chl pool.

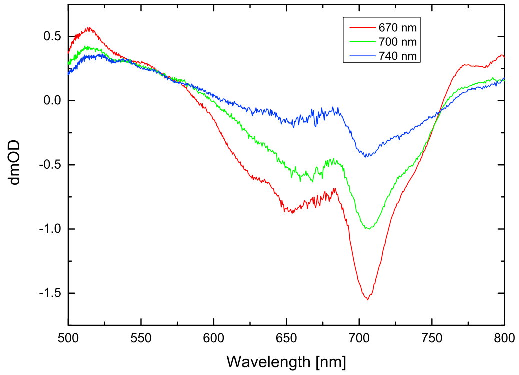

FIGURE 8 Spectra obtained at 1.5 ns for the FRL-PSI sample at pump wavelengths of 670 nm, 700 nm, and 740 nm. To see this figure in color, go online.

The assignment of the third compartment (compartment C) depends on the assumptions made about the charge-separation rate. If slower charge separation is assumed, compartment C should represent the RC* state, and the next transition, with a 39 ps time constant, should lead to the $P700^{+\bullet}$ $A_1^{-\bullet}$ state (compartment D). This variant is shown in Fig. 11 *a1*.

On the other hand, if the charge separation happens in <1 ps, compartment C should correspond to the first radical pair. Based on published evidence, the first radical pair, RP1, could be either $A^{+\bullet}A_0^{-\bullet}$ (6,9) or $P700^{+\bullet}A_0^{-\bullet}$ (19,28). Although the visible pump-probe data cannot definitively distinguish between either of the possibilities, an assignment to $P700^{+\bullet}$ $A_0^{-\bullet}$ may be argued, which in the subsequent step evolves into $P700^{+\bullet}$ $A_1^{-\bullet}$ (RP2), whereas the RC* state is not resolved (Fig. 11 *a2*).

As can be seen from Fig. 10 *a*, compartment C has a much broader bleach than, for example, compartment B. It also appears to consist of two overlapping bands: one centered at ~690 nm and the other at 706 nm. This seems to suggest that the two Chl pools contribute to compartment C. Chauvet et al. (31) showed that the $A_0^{-\bullet}/A_0$ spectrum has a bleach at 684 nm. This is one possible candidate for a member of compartment C. However, the positive band at 650 nm could be taken as an indication of excited-state absorption, rather than an assignment to a radical pair. However, the $A_0^{-\bullet}/A_0$ spectrum in Ref. (31) also has a positive band in this region, and a similar band was assigned to $A_0^{-\bullet}/A_0$ by Shelaev et al. (27). Furthermore, the compartment C spectrum has a positive signal in the 750–780 nm region, which is assigned to $P700^{+\bullet}$-induced absorption (10) and is consistent with the kinetics and wavelengths observed in *C. reinhardtii* as well as *Synechocystis* sp. 6803 PSI (25,31).

The results for 700 nm pump excitation differ substantially from the measurements made with 670 nm pump excitation. The first equilibration stage has a lifetime similar to that of the 0.16 ps compartment of the 670 nm excitation data; however, the second compartment decays with a 10 ps lifetime, versus the 1.5 ps observed in the 670 nm

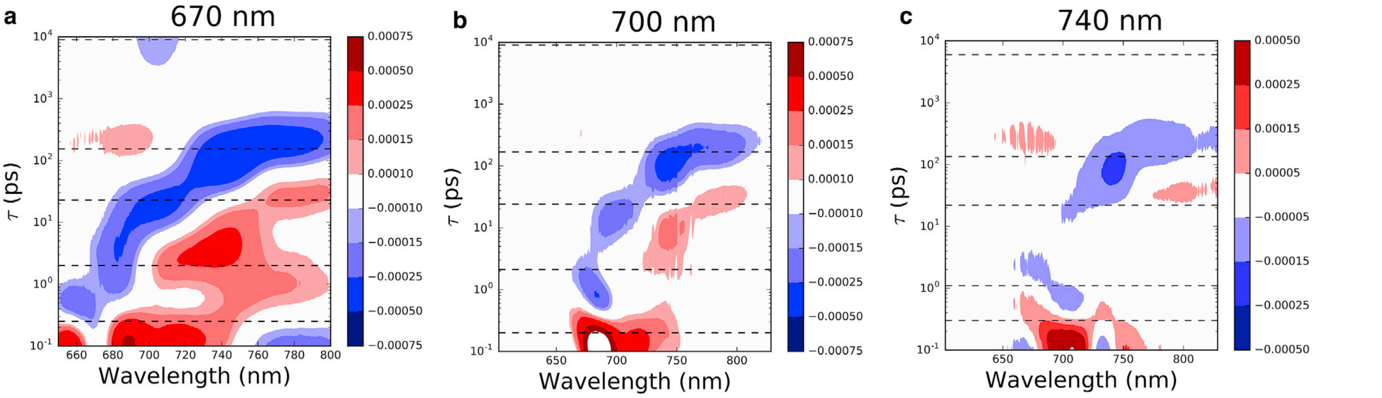

FIGURE 9 (*a–c*) Lifetime-density maps for FRL-PSI with pump wavelengths of (*a*) 670, (*b*) 700, and (*c*) 740 nm. Dashed lines indicate lifetimes observed in sequential model fits (see "Fit results" section). To see this figure in color, go online.

pump results. There is also a significant difference between the lifetimes of the third compartments: 97 ps with 700 nm pump excitation as opposed to 39 ps with 670 nm excitation. Furthermore, the spectrum of the third compartment does not consist of the same two constituents as with 670 nm excitation. Most of the bleach is at longer wavelengths than 700 nm, with a peak at 710 nm.

The 700 nm excitation is expected to be absorbed more selectively by the Chls that are close to or part of the Chls involved in charge separation. Thus, one would expect the transit times to the charge-separation stage to be faster with 700 nm excitation than with 670 nm excitation. Therefore, we conclude that compartment B represents the state of the charge-separating Chls (Fig. 10 *b*). As with 670 nm excitation, there are two possible assignments of this compartment depending on what value is taken for the intrinsic rate of the charge separation.

If a slow charge-separation rate is assumed, the compartment B spectrum would be assigned to the excited state of the charge-separating Chls (Fig. 11 *b1*). In that case, the compartment B with 700 nm excitation (Figure 10 b) could be compared to the compartment C for the 670 nm excitation data (Figure 10 *a*). The spectrum of the 670 nm pumped compartment C contains a bleach at a shorter wavelength (~688 nm) in addition to one at ~706 nm which could correspond to the major contribution seen in compartment C in the 700 pumped data.

With the 700 nm excitation data, compartment B has also a broad spectrum, but most of the bleach is at wavelengths longer than 700 nm. One possible explanation is that the RC* state has different distributions of the excitation energy among the RC pigments. It is possible that the 700 nm pump causes most of this energy initially to be located around P700 Chls. On the other hand, when the excitation energy reaches the charge-separating pigments in the RC through transfer from the antenna, the recipient of this energy could be preferentially either A or A_0, rather than the P700 Chls. The exact nature of this excitation transfer and the role of the connecting Chls (38) still have not been determined unequivocally. However, A_0 is expected to have absorption at shorter wavelengths than P700 (31), which could explain the prevalence of the 690 nm constituent in the RC* data for the 670 nm excitation.

The alternative interpretation assumes that the charge-separation rate is ultrafast, in which case compartment B should be composed of a mixture of the RC* state and the primary radical pair state (Fig. 11 *b2*).

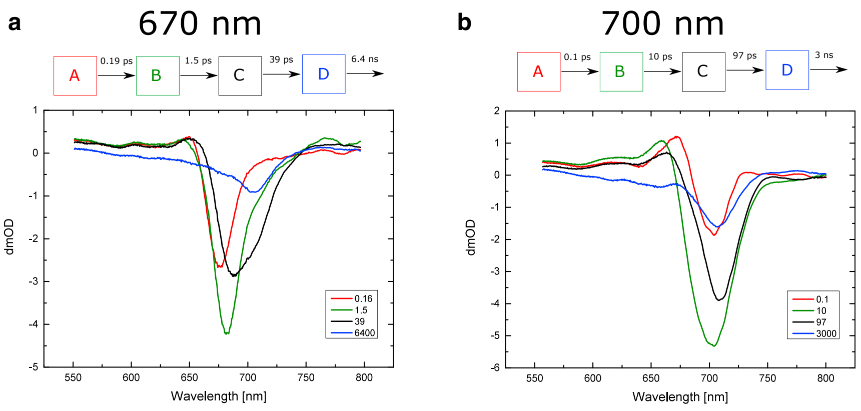

FIGURE 10 (*a* and *b*) Results of sequential fits of WL-PSI data for pump wavelengths of (*a*) 670 nm and (*b*) 700 nm. Above each figure a representation of the model is shown where the color of the box corresponds to the same color spectrum below. To see this figure in color, go online.

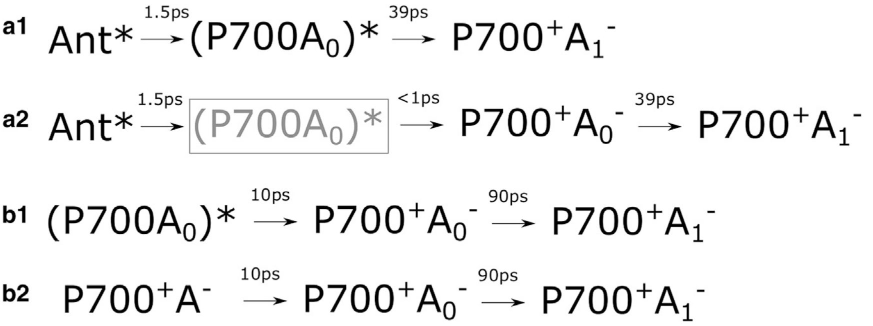

FIGURE 11 (*a1–b2*) Possible contributors to the compartments in the WL-PSI sequential model results for 670 nm excitation (*a1* and *a2*) and 700 nm excitation (*b1* and *b2*). Abbreviations are the same as in Fig. 1. See text for suggested assignment of the primary radical pair to $P700^{+\bullet}A_0^{-\bullet}$ and further details. In the (*a2*) case, ($P700\ A_0$)* is gray because this state could be present but not resolved spectrally in the current data.

For either model assuming fast or slow charge separation, the third compartment (compartment C) should represent the further evolution of the radical pair, most likely $P700^{+\bullet}$ $A_0^{-\bullet}$, that transitions into the secondary radical pair, $P700^{+\bullet}$ $A_1^{-\bullet}$, represented by compartment D (Fig. 10 *b*). However, the associated difference spectrum for compartment D retains very weak additional features of excited Chls, as seen from some remaining excited-state absorption at ~670 nm, so it could still represent a mixture of excited pigments and charge separation.

Furthermore, it is seen that the last compartments for both pump wavelengths have very similar spectra, with the main bleach at 706 nm and a 600–680 nm shoulder on the blue side of the main bleach. This compartment can be assigned to $P700^{+\bullet}$ $A_1^{-\bullet}$ (27).

The WL-PSI TA measurements are generally comparable to those reported in previous studies of Chl *a*-containing PSI from various sources (6,9,18,23,25,30,31). The global analysis that uses homogeneous transformation does not separate the complex energy transfer and charge-separation processes in PSI particles (Fig. 10). This is also demonstrated by the lifetime maps originally developed by Holzwarth et al. (30), which may guide the application and development of more elaborate models. Since the sequential model resulted in compartments of mixed character, we next used target modeling, as discussed further below.

FRL sequential fitting results. Fig. 12 shows the results of the sequential fit for the FRL-PSI data, using a limiting number of compartments as chosen from resulting fitting statistics and residuals.

A key observation is made for 740 nm excitation, which shows that direct excitation of Chl *f* results in charge separation after slow decay and trapping (Fig. 12 *c*).

A four-compartment sequential model was used to fit this data. After the initial equilibration, compartment B has a spectrum with the main bleach at 740 nm corresponding to the Chl *f* main absorption band. As 740 nm excitation is directly absorbed by Chl *f* pigments, it is also shown that compartments B and C also represent signals originating from Chl *f* pigments. However, the last compartment, compartment D, is almost identical to the last compartments in the WL-PSI results, which were assigned to the $P700^{+\bullet}$ $A_1^{-\bullet}$ radical pair, with either 670 nm or 700 nm excitation (Fig. 10, *a* and *b*). This observation leads to the important conclusion that the Chls *f* in FRL-PSI are well connected to or integrated into the charge-separating Chls. The excited energy from Chl *f* can be 1) transferred to the RC and used to form the radical pair with good efficiency, 2) used directly for charge separation, or 3) both, with good efficiency. When we compared the amplitudes of the initial bleach and that of the final spectrum representing the $P700^{+\bullet}$ $A_1^{-\bullet}$ radical

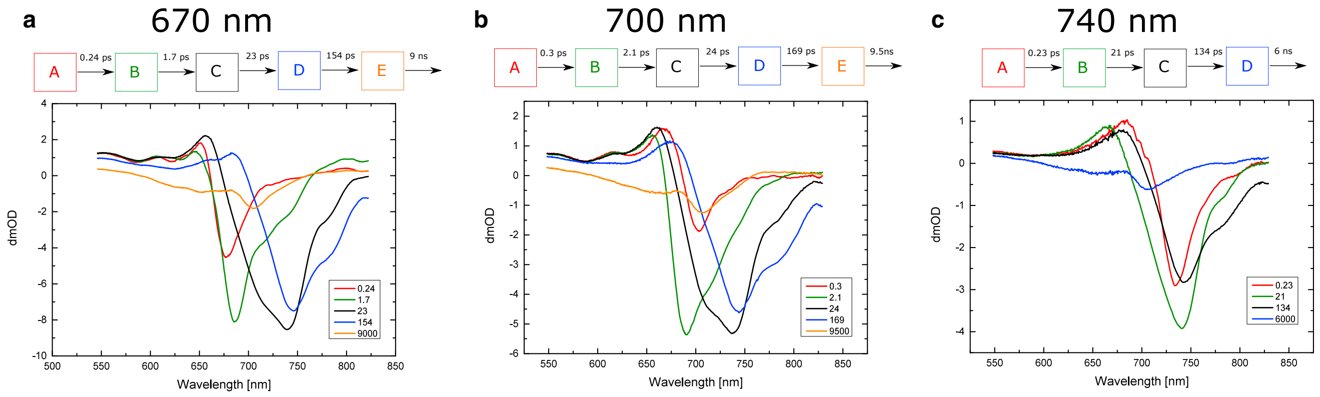

FIGURE 12 (*a–c*) Results of sequential fits of FRL-PSI data with pump wavelengths of (*a*) 670 nm, (*b*) 700 nm, and (*c*) 740 nm. To see this figure in color, go online.

pair, we found they were very similar for both WL-PSI and FRL-PSI (Figs. 10 and 12).

A five-compartment model had to be used to fit the 670 nm and 700 nm pump excitation data, and the results are very similar for both. The first equilibration stage is followed by a compartment that has a major bleach at 685–690 nm and decays with a ~2 ps time constant for both pump wavelengths. The bleach maximum position suggests that this state is located predominantly on Chl *a*, although the emergence of a bleach at 740 nm is also noticeable.

The spectra of the next two compartments, C and D, show significant shifts of the main bleach from the 680 nm region to 740 nm. The third compartment, C, which decays with a 23 ps constant also has a broad shoulder at 680–730 nm as well as small side band at 780 nm. The 740 nm bleach position is consistent with the assignment of this compartment to Chl *f*.

There are two opposing models that can explain these results. The first assumes that the Chl *f* pigments plays an antenna role and the 2 ps transition time from compartment B to compartment C represents downhill transfer of the excited energy toward the Chl *f* (Fig. 13 *c1*). The subsequent two transitions would then describe the evolution of the Chl *f* excited state and the transfer of the excitation to the Chls involved in charge separation. The absence of the Chl *f* in the final radical pair, $P700^{+\bullet}$ $A_1^{-\bullet}$, is supported by the fact that the compartment E spectrum (Fig. 12, *a* and *b*) is almost exactly the same as that of compartment D in the WL-PSI data (Fig. 10, *a* and *b*). Thus, the same final state is reached even if intermediate states involve Chl *f*. Furthermore, this indicates that Chl *f* pigments are well connected and contribute substantially to energy transfer to the charge-separating Chls. The slow transfer times to the RC can be explained by the energy barrier for the transition from Chl *f* to the charge-separating pigments.

The spectra of compartment D using 670 nm and 700 nm excitation (Fig. 12, *a* and *b*) show no bleach in the 690–700 nm region, and instead have a strong bleach at 743 nm. This suggests that most, if not all, of the excitation energy had been transferred to the Chl *f* at this stage. However, the percentage of Chl *f* molecules present in each FRL-PSI core is estimated to be ~8% (see the absorption spectrum in Fig. 2). The fact that this low amount of pigment traps most of the excitation points to an important role of Chl *f* among the core RC pigments.

The second possible model considers the possibility that one or two Chls *f* are located among the charge-separating Chls. Fig. 13, *c2* and *c3*, show an example of substituting Chl *a* for Chl *f* in A_0. According to this model, the 2 ps compartment B decay time is similar to that observed in the WL-PSI 670 pump sequential fit, and would likely represent energy transfer from the antenna to the RC.

As discussed above for the WL-PSI sequential fit, compartment C with 670 nm or 700 nm excitation (Fig. 12, *a* and *b*) can be assigned to 1) the excited state of the charge-separating Chls, RC* (Fig. 13 *c2*), or 2) a mixture of RC* and the first radical pair state (Fig. 13 *c3*), if we assume charge separation to be slow or fast, respectively.

The fourth compartment, D (Fig. 12, *a* and *b*), can clearly be assigned to the further evolution of the primary radical pair for either slow or fast intrinsic charge separation. The spectrum of this compartment shows a dominant bleach at 745 nm that corresponds to Chl *f*. If this model is correct, this would suggest that Chl *f* is one of the constituents of the radical pair and has replaced one of the Chl *a* molecules that are involved in charge separation. It is most commonly assumed that the primary radical pair anion is $A_0^{-\bullet}$ (2); thus, according to this model, Chl *f* could have substituted A_0 Chl *a* in the FRL-PSI RC. Alternatively, since it seems likely that the A Chl is involved in primary charge separation either as the primary acceptor, with $P700^{+\bullet}A^{-\bullet}$ as the primary radical pair, or as the primary donor, with $A^{+\bullet}A_0^{-\bullet}$ as the primary radical pair (6,9), the replacement of Chl *a* at A for a Chl *f* is also a possibility. A decay time of ~160 ps was not observed in WL-PSI in this study, but was seen in *Chlamydomonas* PSI TA lifetime maps produced by Müller et al. (25).

The spectra of compartments E (with 670 nm or 700 nm) and D (with 740 nm excitation) in the FRL-PSI data are almost exactly the same for all pump wavelengths and are also the same as in the WL-PSI data (Fig. 12). This strongly suggests that the final state of PSI charge separation at 1.5 ns after excitation is $P700^{+\bullet}A_1^{-\bullet}$, and that this is the same for both WL- and FRL-grown PSI and is independent of the excitation wavelength.

Target analysis fit results

We tested different target models for the current data sets and assessed the quality of fit for each one. We applied the indicators for fit quality as described by Müller et al.

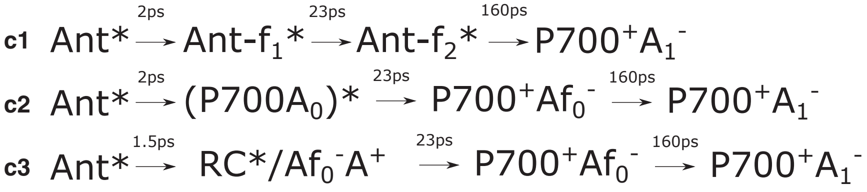

FIGURE 13 Possible contributors to the compartments in the FRL-PSI sequential model results for 670 nm and 700 nm excitation. Abbreviations are the same as in Fig. 1, with Ant-*f* and A*f*$_0$ denoting Chl *f* pigments in the antenna and replacing A_0 correspondingly. See text for more details.

(25) and present the results for one set of target models. Our main goal in using these models was to determine the role of Chl *f* in FRL-PSI. The results of several other target models are provided in the Supporting Materials and Methods. For example, one model (model 5) included a compartment that was equilibrated with the bulk antenna, as proposed by Di Donato et al. (9), who assigned this compartment to the red Chl pool.

Target modeling of WL-PSI TA

The model and the resulting species-associated difference spectra (SADS) for pump wavelengths of 670 nm are presented in Fig. 14 (see Supporting Materials and Methods for concentration profiles).

The spectrum of the first compartment (compartment A) in 670 nm SADS closely resembles the spectrum assigned to antennae in Ref. (25) (see Fig. 1 in (25)). The first lifetime of 0.16 ps represents the energy equilibration, and thus compartment B with a strong bleach at 683 nm represents the equilibrated version of compartment A. As discussed above, compartment B most likely corresponds to the excited state of the antenna Chls.

We propose to use a target model in which the excitation from compartment B is split between two compartments, C and D, as shown in the model in Fig. 14. The proposed branching is based on the spectral shape of compartment C in Fig. 10 *a*, which shows a contribution corresponding

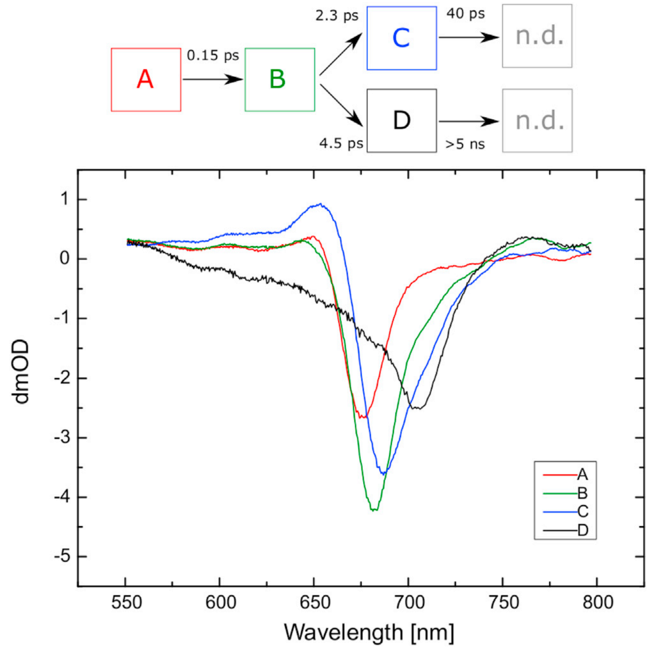

FIGURE 14 A selected kinetic model and the resulting SADS and time constants for the WL-PSI 670 nm pump data. The same target model could not be applied to the 700 nm pump data, and the sequential model was deemed to be best for these data (Fig. 10 *b*). Compartments C and D decay to the millisecond spectrum (Fig. 4); n.d., nondecaying spectrum. The color of each box in the model corresponds to the same color spectrum in the plot. To see this figure in color, go online.

to the long-lived nanosecond spectrum. A spectrum very similar to the spectrum for compartment D is resolved in Fig. 14.

The assignment of compartment D is discussed further in the Discussion section. The time constant for transfer into compartment D is 4 ps, which agrees well with the 4–6 ps peak observed in the lifetime-density map for these data. This lifetime is consistent with the equilibration times between antennae and red-shifted Chls observed by Slavov et al. (8). However, it is also similar to the rate assigned to the transition from the excited state of the charge-separating Chls to the first radical pair by Müller et al. (25) (see Fig. 1).

If the red-shifted Chls *a* absorbing near ~710 nm as seen in the absorption spectrum (Fig. 2, WL) are considered for assignment to compartment D (Fig. 14), then the energy is trapped there for at least several nanoseconds according to the fit results. This contradicts the previously suggested models of red Chl connectivity (8,9). Those models usually assume that red Chl pools are equilibrated with the bulk antenna and lose all excitation on the 10 ps timescale. Therefore, the current results do not strongly support the assignment of compartment D to the red Chl pool.

On the other hand, if compartment D represents the P700 Chls, the most likely assignment would be to the $P700^{+\bullet}$ cation, as its shape remains unchanged until the 2 ns spectrum.

In this case, compartment C could correspond to the anion component of the radical pair. As discussed above, there are two proposed rates of charge separation. In the case of slow charge separation, compartment C should represent the excited state of the charge-separating Chls. However, this does not fit with the assignment of compartment D to $P700^{+\bullet}$, as the formation of the cation cannot overtake that of the anion by tens of picoseconds. Thus, this model favors rapid charge separation. This would result in assignment of compartment C to the $A_0^{-\bullet}$ state or its mixture with the excited states of the charge-separating Chls (RC*). Moreover, it decays with a time constant of 40 ps, which is consistent with previously reported transition times for evolution of the first radical pair to the second radical pair (see Fig. 1).

The WL-PSI data obtained with 700 nm excitation did not produce a satisfactory fit using the same or similar target models. Thus, we concluded that it was best to use the sequential fit to present these data.

Target modeling of FRL-PSI TA

We modeled the Chl *f*-containing FRL-PSI TA data using a kinetic scheme very similar to the one we used for the WL-PSI measurements (Fig. 14), with little modification.

The model for the FRL-PSI measurements obtained with 670 nm and 700 nm excitation and the fit results are presented in Fig. 15, *a* and *b*, respectively. The difference between the two excitation wavelengths is very small, and the two results will be discussed together.

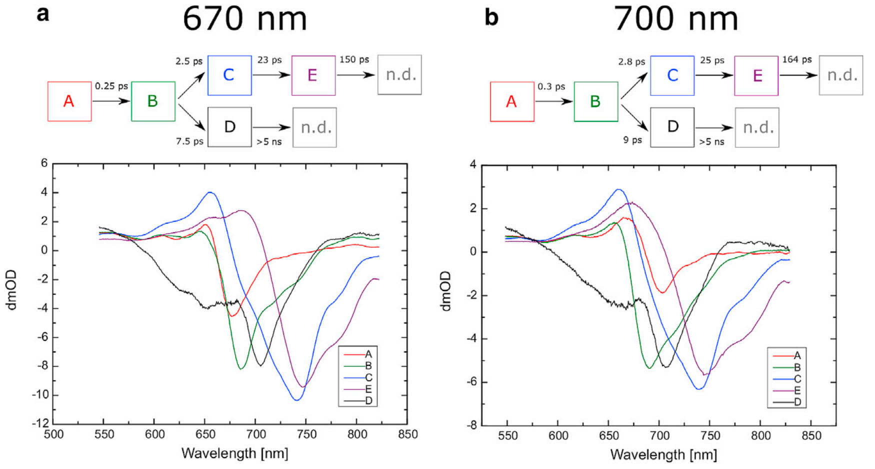

FIGURE 15 (*a* and *b*) Kinetic model and the resulting SADS for FRL-PSI with two pump wavelengths: 670 nm (*a*) and 700 nm (*b*). The 740 nm pump data did not produce a satisfactory fit for a similar model, and a sequential model was deemed to be best for these data. The final compartments, D and E, decay to the millisecond spectrum (Fig. 4). To see this figure in color, go online.

As in the case with WL-PSI, compartment A most likely represents the equilibration within the antenna and decays into compartment B with a subpicosecond lifetime. Consistent with the WL-PSI results, compartment B has a bleach minimum at 685 nm as well as a very similar shape. Thus, it can be assigned to the antenna Chl *a* pigments.

Differences compared with WL-PSI start to emerge in the next step, where the model suggests that the energy is split into two branches.

Compartment D has the same spectrum as in the WL-PSI target analysis results. However, compared with the WL-PSI 670 nm excitation results, the transition time to this compartment increases from 4.5 ps to 7.5–9 ps. Furthermore, the FRL-PSI compartment D kinetics is very similar for both 670 nm and 700 nm excitation. This contrasts with a substantial difference in the compartment decay kinetics observed for the WL-PSI. Furthermore, the spectrum belonging to compartment C with both 670 nm and 700 nm excitation includes a bleach position near 706 nm corresponding to the final nanosecond spectrum E, suggesting the formation of $P700^{+\bullet}$ with an ~2 ps time constant and Chl *a* excitation (Fig. 12, *a* and *b*).

As in the WL-PSI target fitting, compartment D most likely represents the $P700^{+\bullet}$ state. The same spectral shape and position suggests that this state is not affected by the presence of Chl *f*.

The assignment of compartments C and E depends on the chosen model. As discussed in above (Fig. 12), we propose two models that differ in their assumption about the location of the Chl *f* in the RC.

The first model assumes that Chls *f* are in the antenna and thus transition from compartment B to C, indicating funneling of excited energy in the antenna toward Chl *f*. As in the sequential fit, the next two compartments represent the evolution of the Chl *f* excited state and transfer of the excitation to the RC. The branching model assumes a competition between trapping by charge separation, resulting in compartment D (Fig. 15, *a* and *b*) and downhill energy transfer to Chl *f*, corresponding to compartment E. The C-to-E transfer would correspond to a further downhill energy transfer between two Chl *f* pigments with a ~25 ps time constant, where the ~150 ps decay of the final state is caused by a secondary charge separation with a corresponding uphill transfer, as is also seen with direct 740 nm excitation (Fig. 12 *c*). A key feature of this model is that exciton escape from a trapped radical state in compartment D would be too slow for a further downhill energy transfer to Chl *f* in compartment E (Fig. 15).

On the other hand, if Chl *f* is among the charge-separating pigments, compartments C and E would represent evolution of the RC* excited state and formation of the radical pair. According to this model, compartment E should be assigned to a constituent of the primary radical pair $A_0^{-\bullet}$. We propose, however, that the former model is more in line with the expected downhill energy transfer from Chl *a* to Chl *f* and the corresponding long excited-state lifetime of those red states.

Interestingly, the 740 nm pump data did not produce a satisfactory fit using similar target models, and a sequential fit was deemed best to present the data. As the same observation was made with the WL-PSI 700 nm data, it appears that both WL and FRL data with red excitation are best represented with homogeneous kinetics.

DISCUSSION

C. thermalis PCC 7203 WL-PSI results compared with previous studies of PSI

It is worthwhile to compare the WL *C. thermalis* PCC 7203 PSI TA measurements with previously published results for PSI of different origins. Only the WL-PSI measurements can be compared with previously published results, as no previous measurements of Chl *f*-containing PSI have been reported. For 670 nm excitation, the early-stage (up to 1 ps) spectra closely resemble those observed by Gibasiewicz

et al. (22) and Müller et al. (25) for *C. reinhardtii* PSI, except that the spectral bands appear broader due to the larger spectral width of the pump pulse in this work. The major difference that emerges after 1 ps is the development of the shoulder at 706 nm in the *C. thermalis* PCC 7203 WL-PSI data. For *C. reinhardtii* PSI, most of the bleaching is at wavelengths shorter than 700 nm for both 670 nm and 700 nm excitation (see Fig. 2 in Ref. (25)). In contrast, the 706 nm shoulder becomes the dominant spectral feature for delays longer than 100 ps in the *C. thermalis* PCC 7203 WL-PSI data. Furthermore, for the 700 nm excitation, even the early delay data differ substantially from the *C. reinhardtii* PSI results.

Bleaching at wavelengths longer than 700 nm was observed in *Synechocystis* sp. PCC 6803 PSI TA by Shelaev et al. (27), and the negative band at 705 nm was assigned to P700/P700$^{+\bullet}$. However, for *Synechocystis* sp. PCC 6803, Melkozernov et al. (23) concluded that the bleaching at 708 nm is caused by an inhomogeneous pool of red pigments, and assigned a 1.5–2.5 ps lifetime in their data to equilibration between the bulk and antenna and the red Chls.

Below, we discuss the possible origin of the negative band at 706 nm based on what is known in the literature and the observations in the WL-PSI TA data.

Origin of the 706 nm bleach

The 706 nm region of the spectrum is within the characteristic Qy absorption region of Chls. Also considering the relative strength of the 706 nm band, it is assigned to Chl *a* molecules.

Fig. 5 shows a comparison between the steady-state absorption spectrum of the PSI sample and the 1.5 ns TA spectrum of the same sample. The correspondence between the 706 nm band in the TA spectrum and the small shoulder on the red side of the steady-state spectrum is close. It should be noted that if Chl *f* were present in the PSI sample, its absorption would be even further shifted to the red, around 740 nm (see Fig. 2).

On the other hand, for the 670 nm excitation, the absence of the 706 nm band in the spectra before 0.5 ps suggests that the Chls responsible for this band do not absorb the 670 nm pump radiation directly, but are well connected to the bulk of the antenna Chls.

It has been reported in the literature that red-shifted Chl *a* molecules are very common in PSI samples originating from cyanobacteria (8–11). For example, in *T. elongatus* PSI, pools at 708 nm, 715 nm, and 719 nm have been reported (8,10,11). There are no data in the literature about the presence of red-shifted Chls in the PSI from *C. thermalis* PCC 7203, which was used in this study. However, the absorption spectrum shows a shoulder at 710 nm (Fig. 2). Thus, the presence of a pool of such red-shifted Chls connected to the bulk antennae could explain the appearance of the 706 nm band in the subpicosecond stages of spectral development (Fig. 3).

Considering a possible assignment to excited-state pigments of the 706 nm band in the 1.5 ns spectrum (Fig. 5), this would suggest that the energy is trapped in these Chls for a duration that would exceed the typical excited-state lifetimes of Chls in the PSI antennae or those of red-shifted Chls (8).

Alternatively, the 706 nm band could also be assigned to a constituent of the excited RC state P700* or its subsequent evolution into P700$^{+\bullet}$. The latter assignment was proposed by Shelaev et al. (27) for *Synechocystis* sp. PCC 6803 PSI. The appearance of the 706 nm band in the 1.5 ns spectrum is expected after the assignment to the P700$^{+\bullet}$ part of the charge-separated state. However, this assignment may not agree with the observation that the band at 706 nm is different from the 700 nm bleach minimum position observed at 1 ms in the −100 ps spectrum. If both of these bands represent P700$^{+\bullet}$, the shift of the peak position should occur between 2 ns and 1 ms after the excitation pulse. The physical origin of this shift could be electrostatic and result from electron transfer from the A_1 anion to the iron-sulfur centers at 1 ms. It is further noted that the spectrum lacks the characteristic excited-state absorption near ~670 nm, which also supports an assignment to a charge-separated state. Both the nanosecond spectrum and the millisecond spectrum of WL- and FRL-PSI from *Chroococcidiopsis thermalis* PCC 7203 lack the spectral features typically seen in PSI from other species, notably *Synechocystis* PCC6803 (27,28) and *S. elongatus* (10). In addition to a bleach at 703 nm assigned to P700$^{+\bullet}$, another feature is typically seen near 693 nm, and is generally assigned to a neutral Chl *a* close to P700$^{+\bullet}$ (19). Semenov et al. (28) discussed the assignment as developing from a Kerr shift from A_0 in the field of the P700$^{+\bullet}$$A_1^{-\bullet}$ pair or from nearby antenna pigments. For *Synechocystis* PSI, Dashdorj et al. (39) reported that between 200 ps and 1 s, the bandshift feature at 685 nm(−)/693 nm(+) decayed relative to the P700 bleach. Thus, the electrochromic shift was assigned to Chl *a* pigments near the A_1 site (39). The WL- and FRL-PSI from *C. thermalis* PCC 7203 instead show bleach features at ~655 nm in both the 1.5 ns (Fig. 5) and millisecond spectra (Fig. 4), in addition to shift features at 655 nm(−)/675 nm(+) that are at shorter wavelengths but otherwise resemble those reported for *Synechocystis* PCC 6803 PSI (39). As these features are present in both the nanosecond and millisecond spectra, they likely represent an electrochromic shift of relatively blue-absorbing Chl *a* pigments near the P700$^{+\bullet}$ cation (39). We note that both the frequency position and the cross section of the positive feature seen in *Synechocystis* PSI at 690 nm and in *C. thermalis* PCC 7203 PSI at 675 nm are species dependent (40).

Other possible assignments for the 706 nm bleach include dissociated light-harvesting complexes or even Chl *d* present

in the sample. The exact nature of this band cannot be unequivocally determined from the current data and analysis. However, as was shown in previous sections, the role of Chl *f* in PSI excitation transfer can also be assessed without strict assignment of the 706 nm band. The analysis presented in this study favors an assignment of the 706 nm bleach at 2 ns to $P700^{+\bullet}$, in agreement with Shelaev et al. (27). Strong support is provided by the efficient transfer in Chl *f*-containing FRL-PSI to compartment D with excitation at 740 nm (Fig. 12 *c*). An alternative assignment to a radiative state is unlikely, considering the bleach position at 740 nm of its precursor, compartment C.

Possible role of Chl *f* in FRL-PSI

There is no evidence in the results above that the inclusion of Chl *f* in PSI would in any way reduce the quantum yield of light harvesting or even charge separation. On the contrary, for both WL-PSI and FRL-PSI, the 2 ns TA spectra have very similar shapes as well as comparable amplitudes. Moreover, the same is true for the -100 ps TA spectrum, which shows the spectrum of the radical pair 1 ms after excitation (Fig. 4). Furthermore, the FRL-PSI bleaches in the Chl *f* region decay with lifetimes (~150 ps) much faster than would be expected for excited-state decay of isolated Chl *f* pigments (~5 ns) (15), suggesting an assignment to trapping by charge separation rather than fluorescence. All of these results indicate that Chl *f* is an important constituent of the excitation and charge-transfer process in FRL-PSI. It is worth noting that we estimate the relative amount of Chl *a* pigments replaced by Chls *f* to be ~8%. This means that the FRL-PSI contains only a small pool of Chls *f*, with perhaps 10 pigments in total. The strong bleaches in the 740 nm region are due to funneling of the excitation energy toward Chls *f*, and not to the presence of a large number of such pigments.

Although the exact location of the Chls *f* cannot be determined from the TA results presented here, some suggestions can be made. First of all, the same long-lived spectra (2 ns and 1 ms) are observed for 740 nm excitation as for the other excitation wavelengths. Therefore, charge separation can occur efficiently even if Chl *f* molecules are preferentially excited. This suggests that one or more Chls *f* are located in or close to the chain of the pigments that perform charge separation, and that the site energies of the pigments in the direct environment of the Chl *f* molecules will determine the transfer and trapping. In addition, if assignment of the 706 nm band to the $P700^{+\bullet}$ cation is correct, this band stays the same for both WL- and FRL-PSI. This would suggest that Chl *f* does not affect $P700^{+\bullet}$ formation. On the other hand, for FRL-PSI, the bleaches in the 685–700 nm region completely disappear after 10–30 ps and are replaced by the bleaches around 740 nm. This would suggest that the Chls responsible for absorption in this region get replaced by Chl *f*.

Based on the experimental and fitting results, we discuss two possible models for the role of Chl *f* in the PSI processes. According to the first model, Chls *f* are located at a well-connected location in the bulk antenna. They rapidly accumulate the excitation energy and slowly transfer to the RC, where charge separation happens. The rate of the charge-separation process is reduced compared with WL-PSI. However, the data suggest that most, if not all, of the excitation passes through the Chl *f* on route to the RC. This indicates that Chl *f* is likely close to the RC and may serve as a connecting Chl. The spectral features of compartment D, with 670 nm or 700 nm excitation, and compartment C, with 740 nm excitation, include a bleach position near ~775 nm that resembles stimulated emission. Considering the steady-state fluorescence and emission wavelengths up to 800 nm of FRL-PSI at room temperature, an assignment to a radiative species with a 150 ps decay time constant is favored. In this interpretation, a small pool of Chl *f* pigments act as a far-red antenna sink, such that the Franck-Condon factor with the charge-separating Chls is sufficient to allow transfer within the excited-state lifetime of the Chl *f* pigments.

The second possible model suggests that Chls *f* are included in the charge-separating pigments, replacing one or more of the Chl *a* pigments there. If the 706 nm band represents the state of P700 pigments, the most likely candidate for substitution would be A_0. This interpretation includes an assignment of compartments C and D in Fig. 12, *a* and *b*, to a charge-separated state, such that the 2 ps transfer time constant would irreversibly trap all excited-state populations in the Chl *a* antenna pigments. This would require the steady-state emission of Chl *f*-containing FRL-PSI to be significantly reduced compared with Chl *a*-containing WL-PSI. Consequently, we favor an assignment of compartments C and D to a radiative state, and hence an antenna role for at least two Chl *f* pigments. It is possible that Chl *f* is close to P700, for example, in the position of acceptor pigment A, but it is not modeled here explicitly.

In both cases, the evolution of the charge-separated step in Chl *f*-containing PSI appears to be slower than the corresponding process in WL-PSI. The fitting procedure shows a two-step transition (with 25 ps and 160 ps time constants) for FRL-PSI, and a single transition for WL-PSI (with time constants in the range of 24–90 ps depending on the wavelength of excitation). This suggests that the charge separation is slowed down by the presence of Chl *f*, although a quantitative comparison of early and nanosecond bleach amplitudes shows that the charge-separation efficiency is comparable for the WL- and FRL-grown PSI (Supporting Materials and Methods). However, it should be noted that there is a possibility that the 150 ps component could not be resolved for the WL-PSI by the fitting process due to overlap of the spectral features.

The possibility that the 706 nm band corresponds to a red Chl pool can be considered, although assignment to $P700^{+\bullet}$

is better supported. In this case, the data clearly show that Chl *f* would be much better connected to the RC than the red-shifted Chl *a*, and would play a more important role in the charge-separation process. A possible assignment of the 706 nm band to an excited state would also exclude an assignment of compartment C in Fig. 12 *c* to a radiative state.

In summary, we show evidence that with excitation of Chl *f*, charge separation occurs efficiently despite the evident uphill transfer that is required. The kinetics shows a rapid 2 ps time constant for almost complete transfer to Chl *f* if Chl *a* is pumped with a wavelength of 670 nm or 700 nm. The efficiency and rapid kinetics of the energy transfer to the first Chl *f* sink state (compartment C in Fig. 12, *a* and *b*) are remarkable considering the large number of Chl *a* pigments in the PSI particle. Target models can be developed that assume a branching in the formation of the charge separation that applies to blue excitation (670 nm or 700 nm) of either WL- or FRL-PSI.

SUPPORTING MATERIAL

Supporting Materials and Methods, Supporting Results, and six figures are available at http://www.biophysj.org/biophysj/supplemental/S0006-3495(16)34326-0.

AUTHOR CONTRIBUTIONS

J.J.v.T. and A.W.R. designed the research. M.K. and G.D. performed the measurements. D.N. initiated the study of FRL-PSI and prepared the sample. M.K. and G.D. performed the data analysis. J.J.v.T., M.K., and A.W.R. wrote the manuscript.

ACKNOWLEDGMENTS

We thank Andrea Fantuzzi, Alison Telfer, Sephano Santabarbara, and Tanai Cardona for useful discussions.

This work was supported by the Leverhulme Trust through award RPG-2014-126 to J.J.v.T. A.W.R. received support from the Biotechnology and Biological Sciences Research Council through grant BB/L011506/1.

REFERENCES

1. Jordan, P., P. Fromme, …, N. Krauss. 2001. Three-dimensional structure of cyanobacterial photosystem I at 2.5 A resolution. *Nature.* 411:909–917.
2. Brettel, K., and W. Leibl. 2001. Electron transfer in photosystem I. *Biochim. Biophys. Acta.* 1507:100–114.
3. Croce, R., and H. van Amerongen. 2013. Light-harvesting in photosystem I. *Photosynth. Res.* 116:153–166.
4. Joliot, P., and A. Joliot. 1999. In vivo analysis of the electron transfer within photosystem I: are the two phylloquinones involved? *Biochemistry.* 38:11130–11136.
5. Guergova-Kuras, M., B. Boudreaux, …, K. Redding. 2001. Evidence for two active branches for electron transfer in photosystem I. *Proc. Natl. Acad. Sci. USA.* 98:4437–4442.
6. Müller, M. G., C. Slavov, …, A. R. Holzwarth. 2010. Independent initiation of primary electron transfer in the two branches of the photosystem I reaction center. *Proc. Natl. Acad. Sci. USA.* 107:4123–4128.
7. Golbeck, J. H., editor. 2006. Photosystem I. The Light-Driven Plastocyanin: Ferredoxin Oxidoreductase. Dordrecht, The Netherlands.
8. Slavov, C., E. El-Mohsnawy, …, A. R. Holzwarth. 2009. Trapping kinetics in isolated cyanobacterial PS I complexes. *Chem. Phys.* 357:163–170.
9. Di Donato, M., A. D. Stahl, …, M. L. Groot. 2011. Cofactors involved in light-driven charge separation in photosystem I identified by subpicosecond infrared spectroscopy. *Biochemistry.* 50:480–490.
10. Pålsson, L. O., C. Flemming, …, E. Schlodder. 1998. Energy transfer and charge separation in photosystem I: P700 oxidation upon selective excitation of the long-wavelength antenna chlorophylls of Synechococcus elongatus. *Biophys. J.* 74:2611–2622.
11. Zazubovich, V., S. Matsuzaki, …, G. J. Small. 2002. Red antenna states of photosystem I from cyanobacterium Synechococcus elongatus: a spectral hole burning study. *Chem. Phys.* 275:47–59.
12. Schlodder, E., M. Hussels, …, M. Brecht. 2011. Fluorescence of the various red antenna states in photosystem I complexes from cyanobacteria is affected differently by the redox state of P700. *Biochim. Biophys. Acta.* 1807:1423–1431.
13. Schlodder, E., F. Lendzian, …, N. V. Karapetyan. 2014. Long-wavelength limit of photochemical energy conversion in Photosystem I. *J. Am. Chem. Soc.* 136:3904–3918.
14. Chen, M., M. Schliep, …, H. Scheer. 2010. A red-shifted chlorophyll. *Science.* 329:1318–1319.
15. Niedzwiedzki, D. M., H. Liu, …, R. E. Blankenship. 2014. Excited state properties of chlorophyll f in organic solvents at ambient and cryogenic temperatures. *Photosynth. Res.* 121:25–34.
16. Allakhverdiev, S. I., V. D. Kreslavski, …, J.-R. Shen. 2016. Chlorophylls d and f and their role in primary photosynthetic processes of cyanobacteria. *Biochemistry (Mosc.).* 81:201–212.
17. Chen, M. 2014. Chlorophyll modifications and their spectral extension in oxygenic photosynthesis. *Annu. Rev. Biochem.* 83:317–340.
18. Savikhin, S., W. Xu, …, W. S. Struve. 2000. Ultrafast primary processes in PS I from *Synechocystis* sp. PCC 6803: roles of P700 and A(0). *Biophys. J.* 79:1573–1586.
19. Savikhin, S., W. Xu, …, W. S. Struve. 2001. Kinetics of charge separation and A0---> A1 electron transfer in photosystem I reaction centers. *Biochemistry.* 40:9282–9290.
20. Gobets, B., I. H. van Stokkum, …, R. van Grondelle. 2001. Time-resolved fluorescence emission measurements of photosystem I particles of various cyanobacteria: a unified compartmental model. *Biophys. J.* 81:407–424.
21. Gobets, B., I. H. M. van Stokkum, …, R. van Grondelle. 2003. Excitation wavelength dependence of the fluorescence kinetics in Photosystem I particles from *Synechocystis* PCC 6803 and Synechococcus elongatus. *Biophys. J.* 85:3883–3898.
22. Gibasiewicz, K., V. M. Ramesh, …, A. N. Webber. 2001. Excitation dynamics in the core antenna of PS I from Chlamydomonas reinhardtii CC 2696 at room temperature. *J. Phys. Chem. B.* 105:11498–11506.
23. Melkozernov, A. N., S. Lin, and R. E. Blankenship. 2000. Excitation dynamics and heterogeneity of energy equilibration in the core antenna of photosystem I from the cyanobacterium *Synechocystis* sp. PCC 6803. *Biochemistry.* 39:1489–1498.
24. Melkozernov, A. N. 2001. Excitation energy transfer in Photosystem I from oxygenic organisms. *Photosynth. Res.* 70:129–153.
25. Müller, M. G., J. Niklas, …, A. R. Holzwarth. 2003. Ultrafast transient absorption studies on Photosystem I reaction centers from Chlamydomonas reinhardtii. 1. A new interpretation of the energy trapping and early electron transfer steps in Photosystem I. *Biophys. J.* 85:3899–3922.
26. van Stokkum, I. H. M., T. E. Desquilbet, …, B. Robert. 2013. Energy transfer and trapping in red-chlorophyll-free photosystem I from Synechococcus WH 7803. *J. Phys. Chem. B.* 117:11176–11183.

27. Shelaev, I. V., F. E. Gostev, …, A. Y. Semenov. 2010. Femtosecond primary charge separation in *Synechocystis* sp. PCC 6803 photosystem I. *Biochim. Biophys. Acta.* 1797:1410–1420.
28. Semenov, A. Y., I. V. Shelaev, …, V. A. Nadtochenko. 2012. Primary steps of electron and energy transfer in photosystem I: effect of excitation pulse wavelength. *Biochemistry (Mosc.).* 77:1011–1020.
29. Kompanets, V., V. Shubin, …, A. Razjivin. 2014. Red chlorophyll excitation dynamics in Arthrospira platensis photosystem I trimeric complexes as studied by femtosecond transient absorption spectroscopy. *FEBS Lett.* 588:3441–3444.
30. Holzwarth, A. R., M. G. Müller, …, W. Lubitz. 2006. Ultrafast transient absorption studies on photosystem I reaction centers from Chlamydomonas reinhardtii. 2: mutations near the P700 reaction center chlorophylls provide new insight into the nature of the primary electron donor. *Biophys. J.* 90:552–565.
31. Chauvet, A., N. Dashdorj, …, S. Savikhin. 2012. Spectral resolution of the primary electron acceptor A0 in Photosystem I. *J. Phys. Chem. B.* 116:3380–3386.
32. Rippka, R., J. Deruelles, …, R. Y. Stanier. 1979. Generic assignments, strain histories and properties of pure cultures of cyanobacteria. *Microbiology.* 111:1–61.
33. Fitzpatrick, A. E., C. N. Lincoln, …, J. J. van Thor. 2012. Pump-dump-probe and pump-repump-probe ultrafast spectroscopy resolves cross section of an early ground state intermediate and stimulated emission in the photoreactions of the Pr ground state of the cyanobacterial phytochrome Cph1. *J. Phys. Chem. B.* 116:1077–1088.
34. van Stokkum, I. H. M., D. S. Larsen, and R. van Grondelle. 2004. Global and target analysis of time-resolved spectra. *Biochim. Biophys. Acta.* 1657:82–104.
35. Croce, R., M. G. Müller, …, A. R. Holzwarth. 2001. Carotenoid-to-chlorophyll energy transfer in recombinant major light-harvesting complex (LHCII) of higher plants. I. Femtosecond transient absorption measurements. *Biophys. J.* 80:901–915.
36. Snellenburg, J. J., S. P. Laptenok, …, I. H. M. v. Stokkum. 2012. Glotaran: a Java-based graphical user interface for the R package TIMP. *J. Stat. Softw.* 49:1–22.
37. van Wilderen, L. J. G. W., C. N. Lincoln, and J. J. van Thor. 2011. Modelling multi-pulse population dynamics from ultrafast spectroscopy. *PLoS One.* 6:e17373.
38. Gibasiewicz, K., V. M. Ramesh, …, A. N. Webber. 2003. Excitonic interactions in wild-type and mutant PSI reaction centers. *Biophys. J.* 85:2547–2559.
39. Dashdorj, N., W. Xu, …, S. Savikhin. 2004. Electrochromic shift of chlorophyll absorption in photosystem I from *Synechocystis* sp. PCC 6803: a probe of optical and dielectric properties around the secondary electron acceptor. *Biophys. J.* 86:3121–3130.
40. Witt, H., E. Bordignon, …, E. Schlodder. 2003. Species-specific differences of the spectroscopic properties of P700: analysis of the influence of non-conserved amino acid residues by site-directed mutagenesis of photosystem I from Chlamydomonas reinhardtii. *J. Biol. Chem.* 278:46760–46771.

Biophysical Journal, Volume *112*

Supplemental Information

Femtosecond Visible Transient Absorption Spectroscopy of Chlorophyll *f*-Containing Photosystem I

Marius Kaucikas, Dennis Nürnberg, Gabriel Dorlhiac, A. William Rutherford, and Jasper J. van Thor

Femtosecond visible transient absorption spectroscopy of Chlorophyll f-containing Photosystem I

M. Kaucikas, D. Nürnberg , G. Dorlhiac , A. W. Rutherford , J. J. van Thor

Supplementary information

Contents

Different Models for WL PSI with 670 nm excitation 2
Model 1: Sequential model 2
Model 2: Sequential model with reverse reactions 2
Model 3 2
Model 4 3
Model 5 3
Model 6 4
Model 7 4
The Effect of the initial excitation distribution between the compartments 4
Model 1: Sequential model 4
Model 3 5
Model 5 5
Fits for the WL PSI 700nm and FRL data 6
WL PSI 700 nm sequential 6
FRL PSI 670 nm excitation 6
Model FRL-1 6
Model FRL-2 7
Pump energy and sample translation effects 8
Theoretical calculations 8
Experimental tests 8
Estimation of Charge Separation Efficiency 13
References 14

Below we present several models that were applied to the PSI TA data. Model scheme, concentration profiles and SADS are shown for each model. The numbers in the model schemes indicates the rate in (ps^{-1}). If not indicated otherwise, the model assumes that initial excitation is compartment A.

Different Models for WL PSI with 670 nm excitation

Model 1: Sequential model

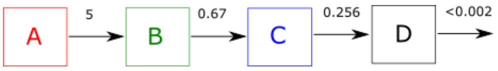

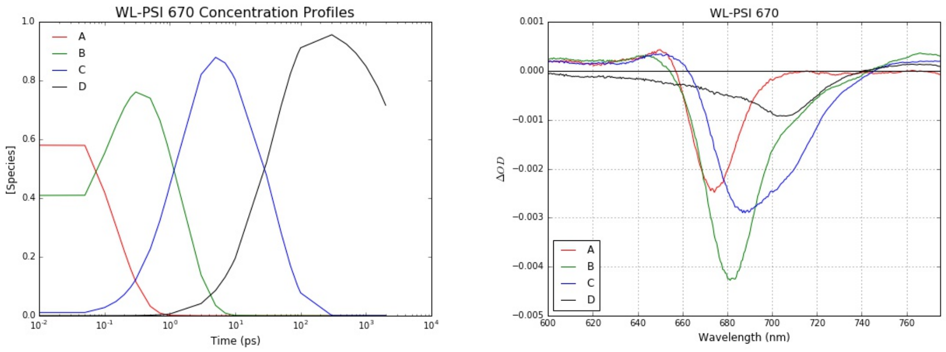

Model 2: Sequential model with reverse reactions

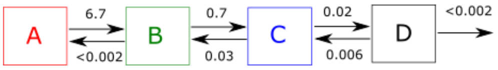

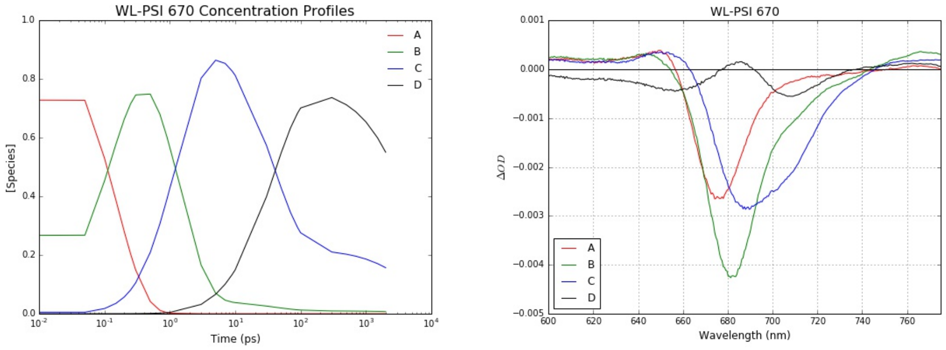

Model 3

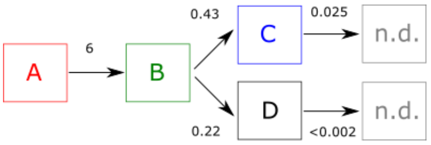

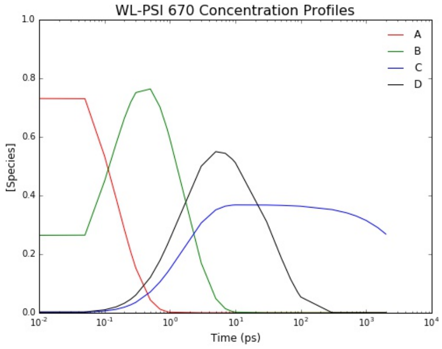

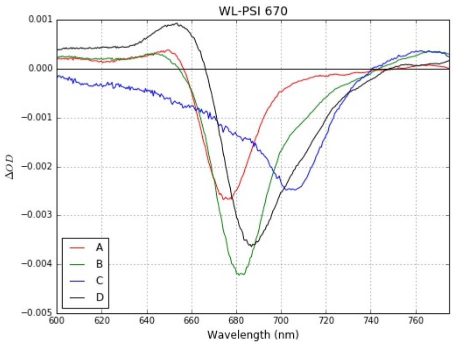

Model 4

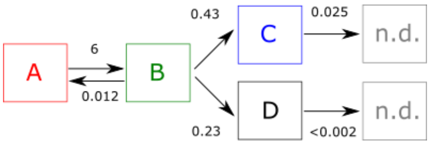

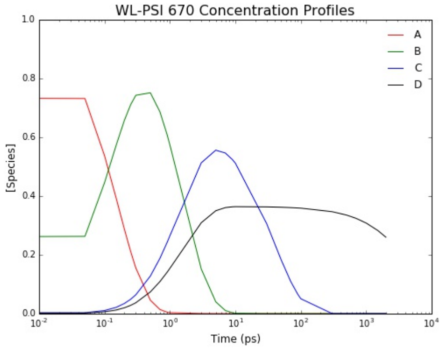

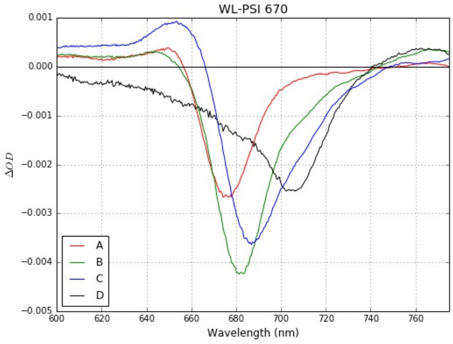

Model 5

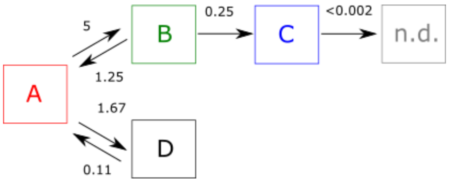

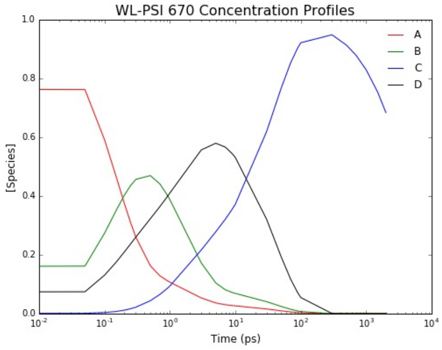

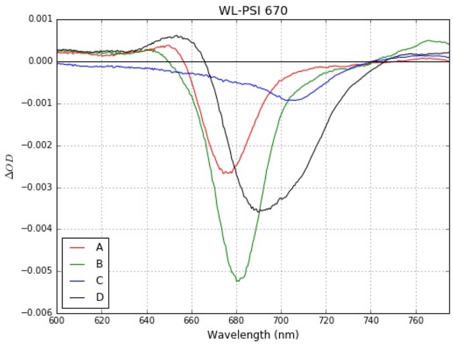

Model 6

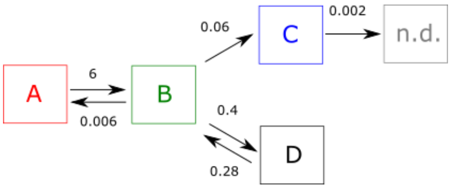

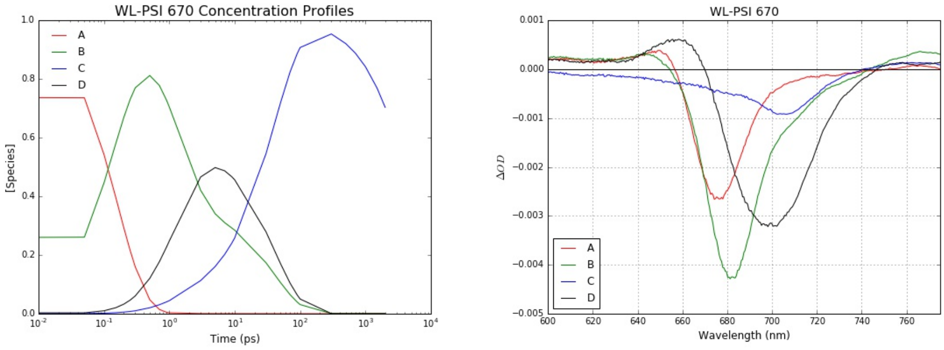

Model 7

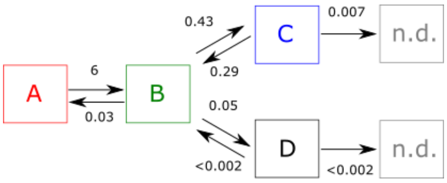

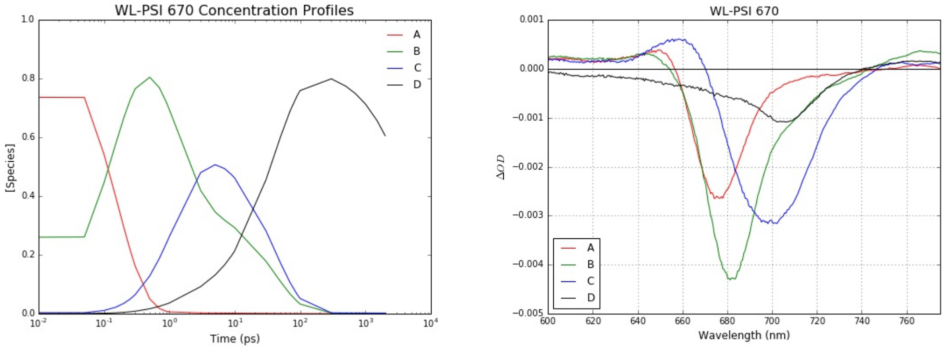

The Effect of the initial excitation distribution between the compartments

Model 1: Sequential model

Initial distribution: 80% A, 20% B

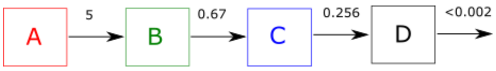

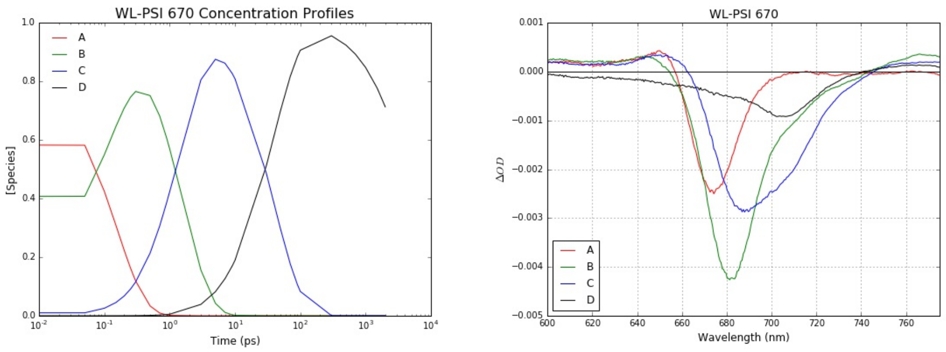

Model 3

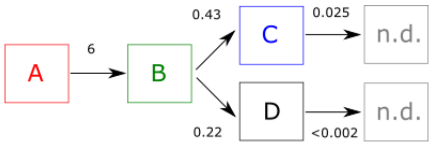

Initial distribution: 80% A, 20% B

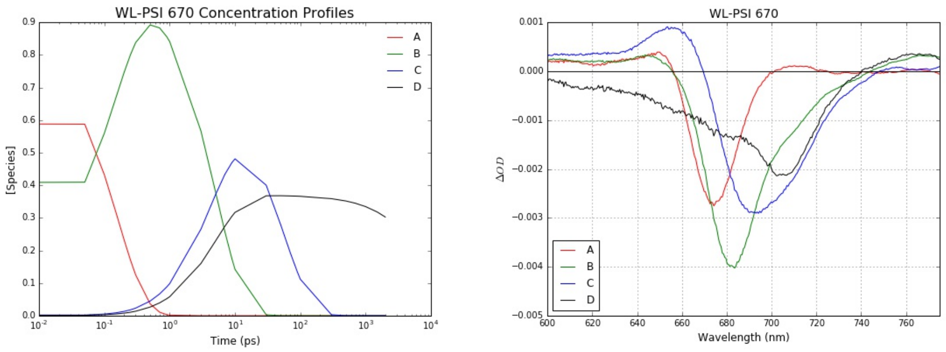

Model 5

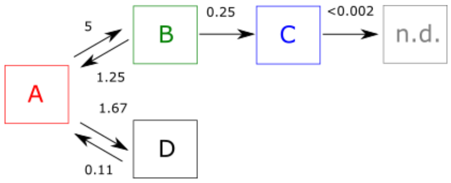

Initial distribution: 80% A, 20% B

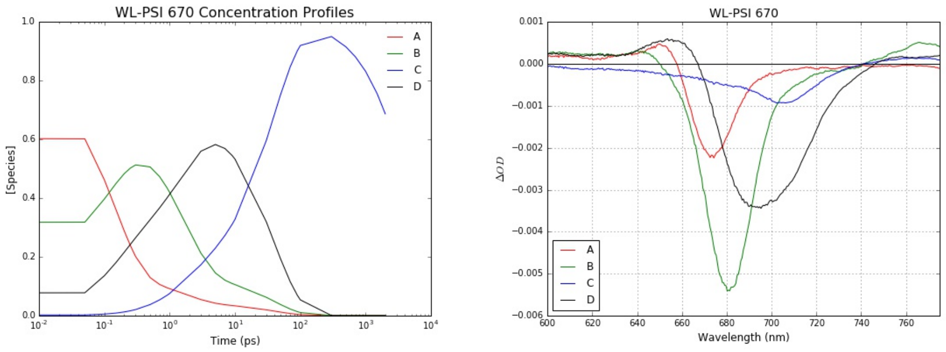

Initial distribution: 45% A, 25% B, 25% D

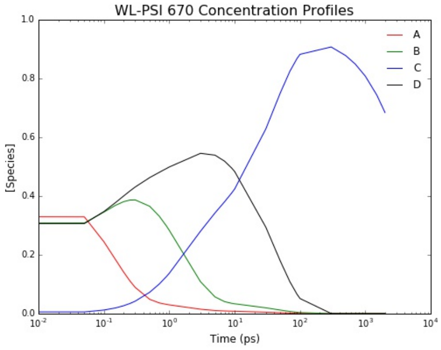

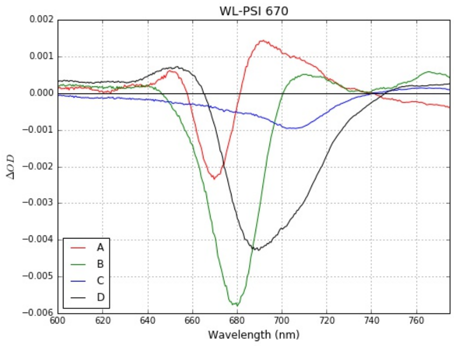

Fits for the WL PSI 700nm and FRL data

WL PSI 700 nm sequential

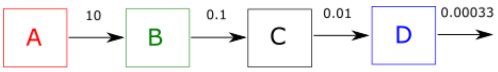

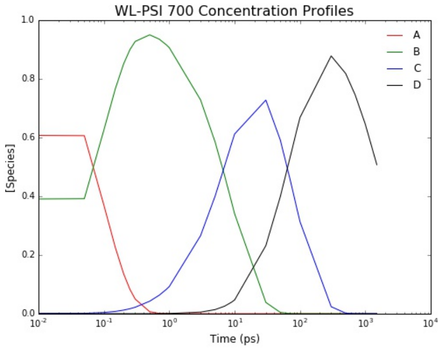

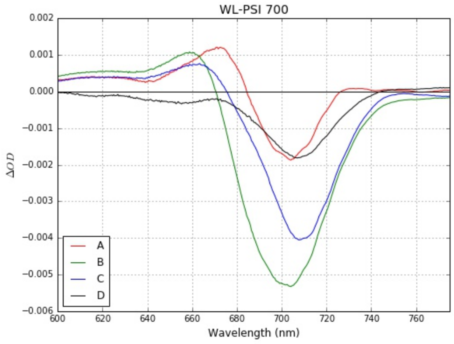

FRL PSI 670 nm excitation

Model FRL-1

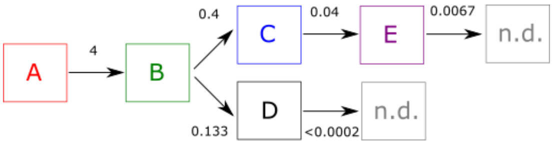

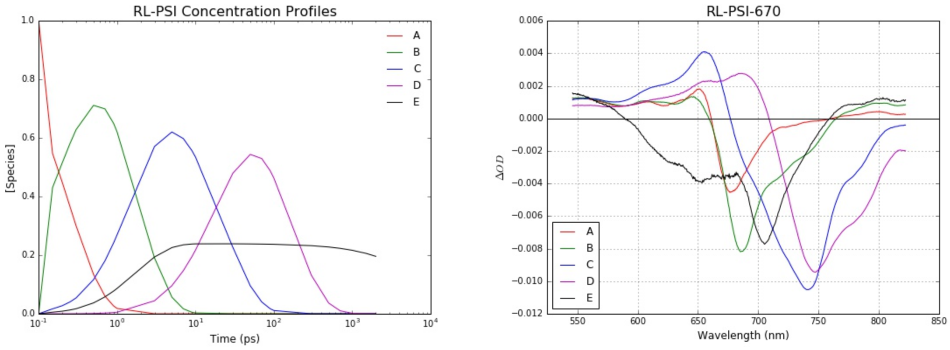
RL-PSI Concentration Profiles
[Species]
Time (ps)
A
B
C
D
E
RL-PSI-670
ΔOD
Wavelength (nm)

Model FRL-2

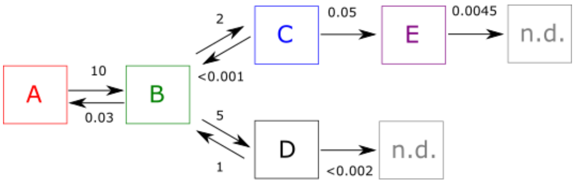
A
10
0.03
B
2
<0.001
5
1
C
0.05
E
0.0045
n.d.
D
<0.002
n.d.

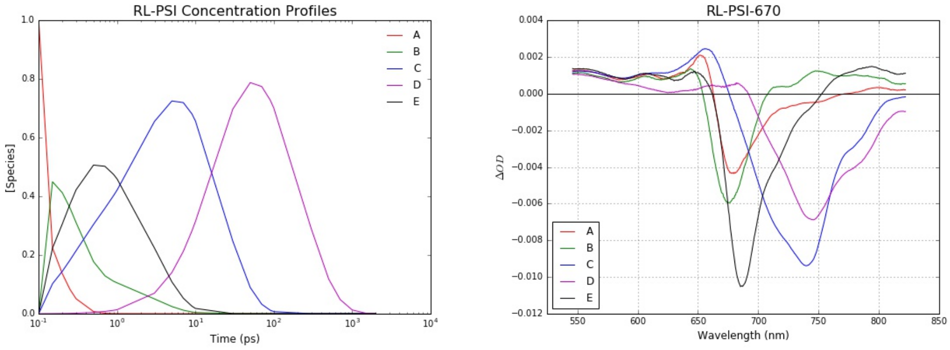
RL-PSI Concentration Profiles
[Species]
Time (ps)
A
B
C
D
E
RL-PSI-670
ΔOD
Wavelength (nm)

Pump energy and sample translation effects

Theoretical calculations

First we attempted theoretical estimation of the photons per particle measure using the model outlined by Müller et al. (1). Although the model relies heavily on knowing the value of the PSIcc extinction coefficient, a reasonable estimate can be obtained using known values for PSI RCs and the number of Chls present in the system. Thus the value of the photons per PSI particle is estimated to be around 1.1. This value indicates that double excitation of single PSI particle is possible and its effect on the resulting spectral kinetics should be investigated.

It should be noted that the photon density as measured in photon count per cm^2 in the measurements reported in this paper is less than 1.4 10^{14} cm^{-2}. This value is higher than approx. 0.14 10^{14} cm^{-2} in Müller et al. paper (2) but comparable to the one in Shleaev et al. paper - 10^{14} cm^{-2} (3) and significantly lower than 19 10^{14} cm^{-2} used by Donato et al. (4).

Recently Yoneda et al. (5) showed that in the case of PSII excitations at 10^{14} cm^{-2} level do not result in significant annihilation processes. While this is not directly applicable to the PSI due to roughly three times higher number of pigments per particle, it does suggest that in order to observe annihilation effects the excitation should exceed 10^{14} cm^{-2} level significantly.

Experimental tests

Additional tests were carried out to access whether the pump energy or the speed of sample translation can influence the results obtained in the measurements described in the main paper. Namely, we looked for the evidence of annihilation effects as well as the presence of “closed” PSI reaction centres.

A sample of FRL PSI was used in these measurements prepared under identical conditions as the one in the original measurements. The pump wavelength was chosen to be 670 nm because it corresponded to the highest sample absorption of all pump wavelengths, resulting in the highest number of absorbed photons. Thus this pump wavelength was most likely to show pump energy related effects. All other conditions were kept exactly the same as in the original measurements, except sample translation speed and pump energy.

The sample translation speed was varied between the stationary sample (indicated by ST=0 in Figs. below) and maximum translation rate allowable by our current setup (ST=10). Typically the measurements reported in the main text were carried out under ST=8-9 translation setting.

Pump energy was varied by introducing neutral density filter in to the pump beam. Three pump pulse energies were used compared to the original value (<10nJ, ND filter with OD=2) – ten times lower (ND filter with OD=3), two times lower (ND filter with OD=2.3) and ten times higher (ND filter with OD=1). Each time the attenuation was changed, the overlap of the pump and probe beams was optimized and time zero was determined.

Sample translation speed

For this test the pump energy was set at the same level as in the main text results. It should be noted that the number of scans recorded in these measurements was approximately 10 times smaller than in the main experiments thus resulting in noisier spectra. Furthermore, the data were collected just at four delay points: -100 ps, 0.5 ps, 3 ps and 2000 ps relative to the pump pulse.

As in Figure S1 the measurements performed at different sample translation speeds result in the spectra of identical shape. However there are some differences in the amplitude of the spectra. To assess these differences we have calculated the ratio of the bleach maximum at 2ns to the bleach maximum in 0.5 ps or 3 ps spectra. These results are shown in Figure S2.

There is a clear trend showing smaller ratios at slower speeds. The trend can be explained by the increasing proportions of the RCs being in the “closed” state. However at high translation speeds, there is little difference between ST=7 and ST=10 speed setting, indeed as smaller decrease occurs. This suggests that the effect of the “closed” RCs does influence the results at this translation speed.

Furthermore information about the presence of the “closed” RCs is contained in the -100 ps spectra (Figure S1, Bottom Right). However due low signal at this delay some smoothing had to be applied before analysis. Furthermore, in order to avoid baseline drift, a difference approach was used to evaluate the size of the bleach at 700 nm. Four reference points on both sides of the bleach maximum were chosen as reference. These points are indicated in the Figure S1, Bottom Right panel by open squares and circles. The size of the bleach was calculated as average of two differences – between bleach maximum and each reference point. The dependence of thus evaluated bleach size on sample translation speed is shown in Figure S3. This Figure also shows how the ratio of -100 ps bleach size to 2 ns bleach size changes with sample translation speed.

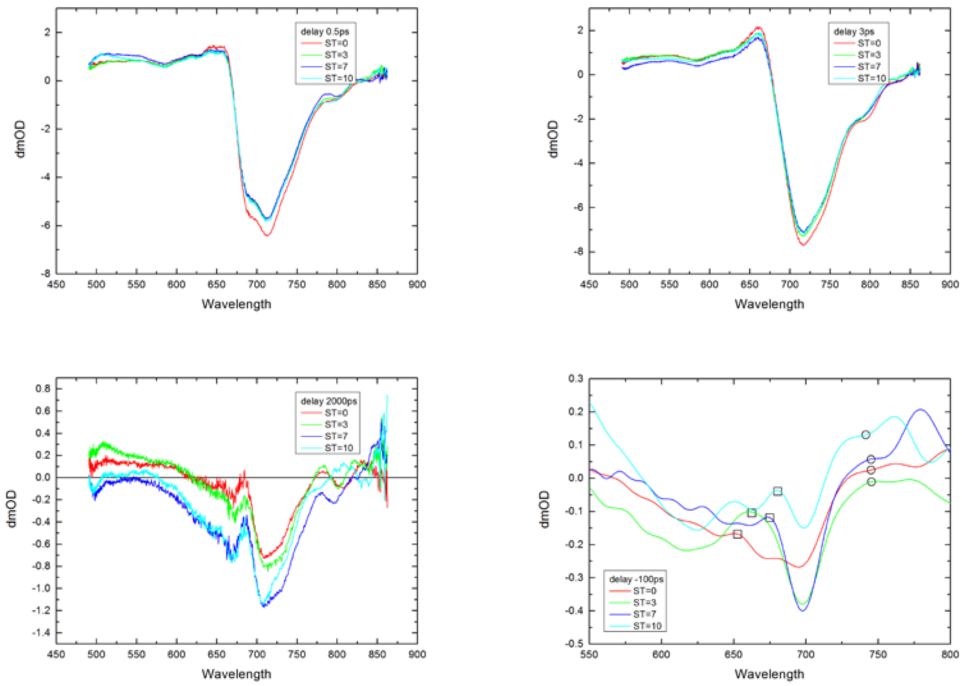

Figure S1 TA difference spectra of FRL PSI for four different sample translation speeds (ST=0,3,7,10) at four different delays: Top Left 0.5 ps, Top Right 3 ps, Bottom Left 2000ps, Bottom Right millisecond (-100 ps, probe before pump). The last spectrum has been smoothed using Loess algorithm and inverted. Open squares and circles indicate the reference levels used to evaluate the size of the bleach at 700 nm.

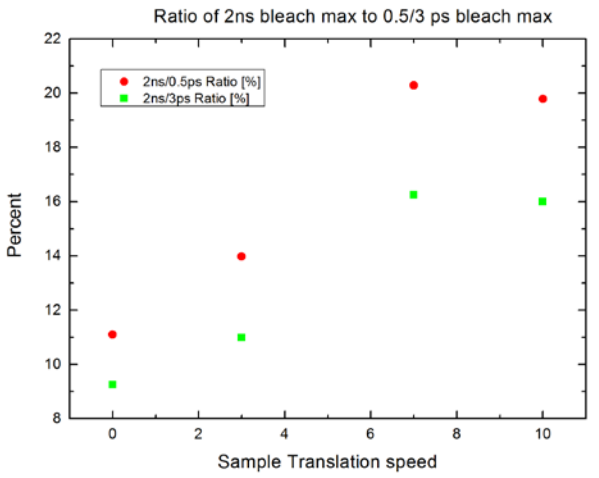

Figure S2 Comparison of the ratio between 2ns bleach and 0.5/3 ps bleach for different sample translaiton speeds

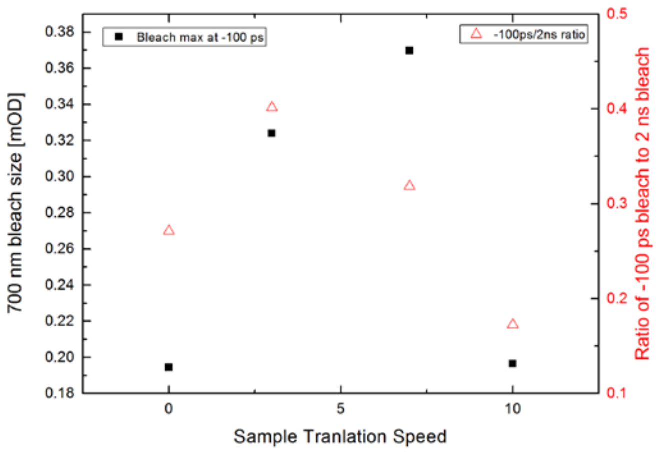

Figure S3 Analysis of the millisecond (-100 ps) spectrum. Black squares (Left scale) show dependence of the 700 nm bleach size on the sample translation speed. The size has been calculated using baseline reference points indicated in Figure above. Red triangles (Right scale) show ratio of millisecond (-100 ps) bleach size to the 2 ns bleach size.

Pump Energy

During these measurements, the sample was translated at the same speed as in the main text result case (ST=7-9) except for the case of the lowest pump energy where due to the low signal ST=1 setting had to be used. Moreover a higher number of scans was averaged in the case of the two lowest energies to produce acceptable signal to noise ratio.

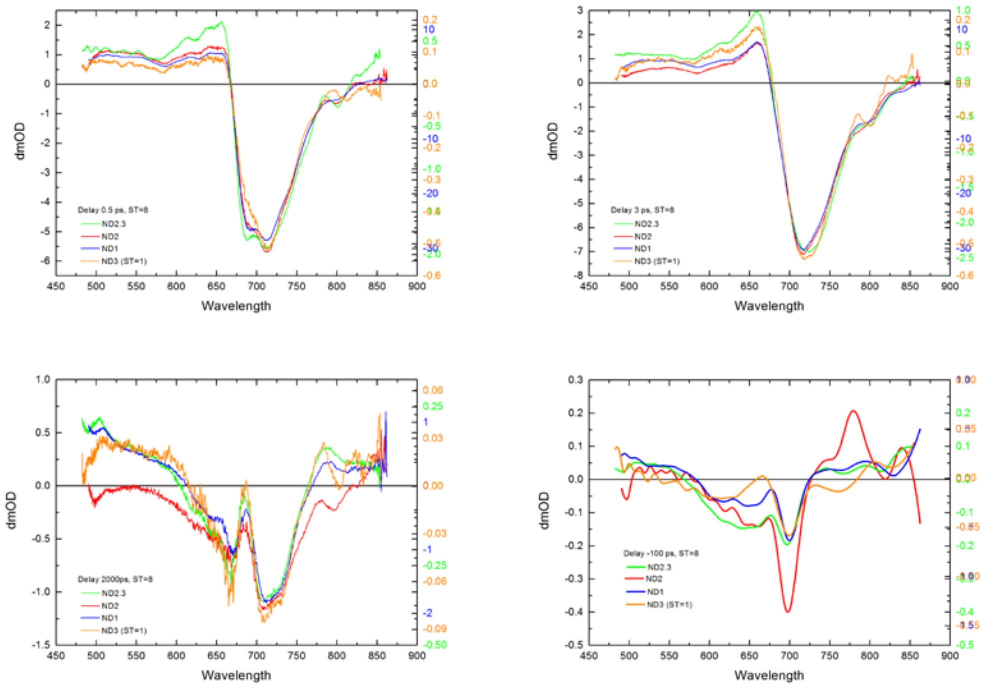

Figure S4 TA difference spectra of FRL PSI for four different pump levels (x0.1, x0.5, x1, x10 relative to the original measurements, indicated in the plot as ND3, ND2.3, ND2 and ND1 correspondingly) at four delays: Top Left 0.5 ps, Top Right 3 ps, Bottom Left 2000 ps, Bottom Right millisecond (-100 ps). The spectra in Bottom Right panel have been smoothed using Loess algorithm and inverted. All spectra are rescaled for comparison purposes.

Figure S4 shows the normalized spectra measured at the four pump energies and at four delay points. It is obvious that the shape of the spectrum stays the same for all pump levels. To assess the effect of the pump energy on the amplitude of the spectra we performed similar analysis as in translation speed case. Namely the ratio of 2 ns bleach maximum and 0.5/3 ps bleach maximum was calculated for all energy levels and is plotted in Figure S5.

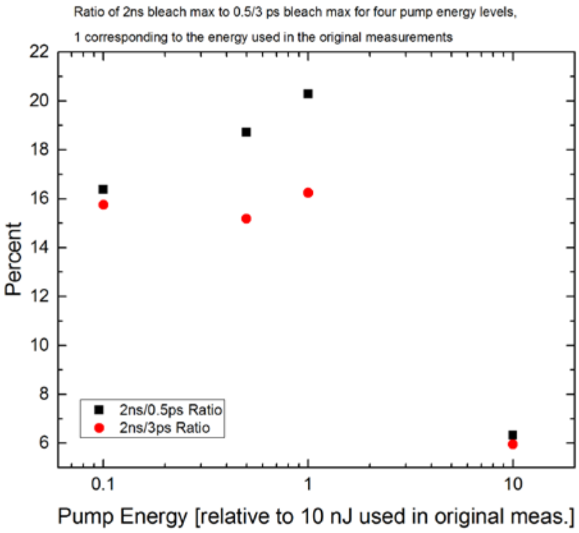

Figure S5 Comparison of the 2ns and 0.5/3 ps bleach ratio for the four pump energy levels.

With a 10-fold increase of pump-power it is clearly seen that this ratio reduces several-fold, indicative of exciton-exciton annihilation. For pulses up to 10-fold weaker we observed a weak trend that reduces the ratio for 2ns/0.5ps bleach amplitudes, but not for the 2ns/3ps (Figure S5). Determination of the 500 fs bleach amplitude may be affected more strongly by the coherent artefact at higher pump powers, therefore considering the 3ps amplitude, we conclude that loss of excited state population through exciton-exciton annihilation is minimal under our experimental conditions.

In conclusion, the experimental tests suggest that the annihilation and "closed" PSI RCs even if present under typical experimental condition used in this work, do not influence the processes of energy and charge transfer and relative yield for radical pair formation substantially.

Estimation of Charge Separation Efficiency

We also attempted to compare the charge separation efficiency between WL and FRL PSI samples. Namely the ratio of the bleach integrals at 2ns and 0.3 ps was used as a measure representative of charge separation efficiency. Figure 6 shows region of the spectra that was used for this estimation.

Surprisingly 670 nm pump data show that the ratio is higher in FRL case – 0.3174 compared to 0.2829 in WL case. The same is true for 700 nm pump – 0.3308 vs. 0.2156.

Although these calculations seem to suggest that CS in FRL PSI is more efficient, in our opinion this type of measure is not reliable enough to make strong conclusions. For example, contribution of still present radiative decay could affect the obtained numbers substantially.

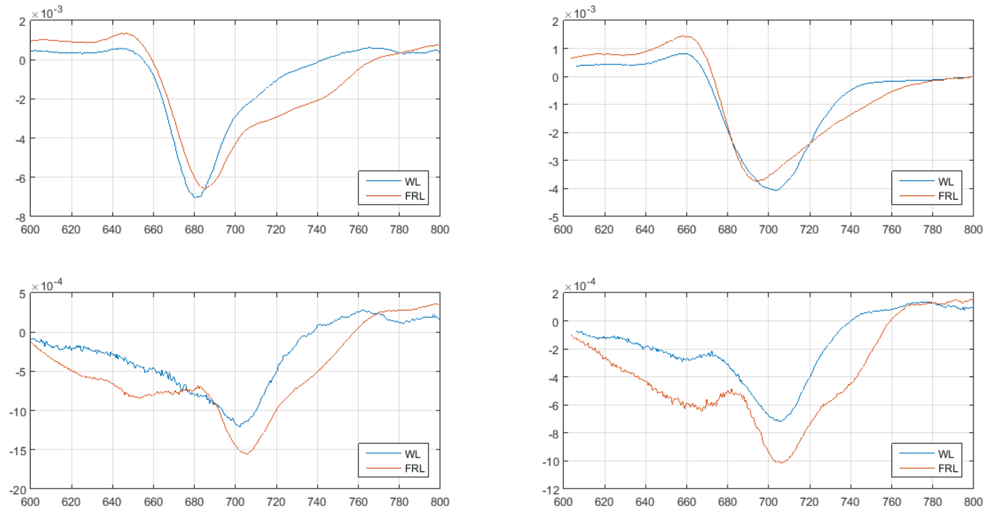

Figure S6 Experimental spectra used in CS efficiency estimation. Left panel 670 nm pump, Right panel 700 nm pump. Top panels show 0.3 ps spectra and Bottom panels 2 ns spectra

References

1. Müller, M.G., M. Hucke, M. Reus, and A.R. Holzwarth. 1996. Annihilation Processes in the Isolated D1-D2-cyt-b559 Reaction Center Complex of Photosystem II. An Intensity-Dependence Study of Femtosecond Transient Absorption † , ‡. J. Phys. Chem. 100: 9537–9544.

2. Müller, M.G., J. Niklas, W. Lubitz, and A.R. Holzwarth. 2003. Ultrafast transient absorption studies on Photosystem I reaction centers from Chlamydomonas reinhardtii. 1. A new interpretation of the energy trapping and early electron transfer steps in Photosystem I. Biophys. J. 85: 3899–922.

3. Shelaev, I. V., F.E. Gostev, M.D. Mamedov, O.M. Sarkisov, V.A. Nadtochenko, V.A. Shuvalov, and A.Y. Semenov. 2010. Femtosecond primary charge separation in Synechocystis sp. PCC 6803 photosystem I. Biochim. Biophys. Acta - Bioenerg. 1797: 1410–1420.

4. Donato, M. Di, A.D. Stahl, I.H.M. Van Stokkum, R. Van Grondelle, and M.L. Groot. 2011. Cofactors involved in light-driven charge separation in photosystem i identified by subpicosecond infrared spectroscopy. Biochemistry. 50: 480–490.

5. Yoneda, Y., T. Katayama, Y. Nagasawa, H. Miyasaka, and Y. Umena. 2016. Dynamics of Excitation Energy Transfer Between the Subunits of Photosystem II Dimer. J. Am. Chem. Soc. : jacs.6b04316.
